# From Plastic Waste to Water Treatment: Floating Magnetic Poly(ethylene Terephthalate)–Fe_3_O_4_/TiO_2_ Photocatalysts for Antidepressant Removal

**DOI:** 10.3390/polym18202555

**Published:** 2026-10-17

**Authors:** Eleni Evgenidou, Nina Maria Ainali, Androniki Rapti, Dimitrios N. Bikiaris, Dimitra A. Lambropoulou

**Affiliations:** 1 Laboratory of Environmental Pollution Control, Department of Chemistry, Aristotle University of Thessaloniki, 541 24 Thessaloniki, Greece; evgenido@chem.auth.gr (E.E.); nsainali@chem.auth.gr (N.M.A.); 2 Centre for Interdisciplinary Research and Innovation (CIRI-AUTH), Balkan Centre, 570 01 Thessaloniki, Greece; 3 Laboratory of Polymer & Color Chemistry and Technology, Department of Chemistry, Aristotle University of Thessaloniki, 541 24 Thessaloniki, Greece; araptia@chem.auth.gr

**Keywords:** photocatalysis, Fe_3_O_4_/TiO_2_ magnetic nanocomposite, recycled PET, PET–Fe_3_O_4_/TiO_2_, floating photocatalyst, beads, antidepressants, water/wastewater treatment, magnetic separation

## Abstract

This study investigates the photocatalytic degradation of a mixture of three widely detected antidepressants, namely venlafaxine, bupropion, and sertraline, using a novel floating magnetic PET–Fe_3_O_4_/TiO_2_ composite photocatalyst. Magnetic Fe_3_O_4_/TiO_2_ nanoparticles were synthesized via co-precipitation/sol–gel and immobilized onto recycled poly(ethylene terephthalate) (PET) beads by non-solvent precipitation. The composites were characterized and evaluated for pharmaceutical removal under simulated solar irradiation, achieving high degradation efficiency and removing the antidepressants within 60–90 min depending on the compound. Performance was strongly influenced by inorganic loading, with the 50 wt% Fe_3_O_4_/TiO_2_ composite giving the highest degradation rates. Water matrix composition also significantly affected degradation kinetics, with lower efficiencies observed in complex environmental matrices due to radical scavenging and competitive adsorption. Although the parent compounds degraded rapidly, complete mineralization required prolonged irradiation, indicating the formation of intermediate transformation products. Scale-up experiments in a continuous-flow pilot reactor showed performance comparable to laboratory-scale results, confirming applicability under realistic operating conditions. The composite beads retained their photocatalytic activity over the two reuse cycles investigated, although some deformation and TiO_2_ loss were observed after reuse. Overall, the floating magnetic PET–Fe_3_O_4_/TiO_2_ photocatalyst offers a sustainable and efficient approach for removing pharmaceutical contaminants from water, combining photocatalytic efficiency with facile recovery, reusability, and valorization of recycled PET waste.

## 1. Introduction

As a result of increased human consumption, a substantial number of pharmaceutical compounds are detected in different water resources. Their entry into the water cycle is, to a great extent, through the wastewater treatment plants (WWTPs) since the latter exhibit poor removal efficiency for many pharmaceutical contaminants. Among the various classes of pharmaceuticals detected in the environment, antidepressants appear to be a frequently detected group of contaminants around the globe, with concentrations ranging from a few ng/L up to low μg/L levels [1]. Their continuous release, which renders them pseudo-persistent contaminants, is mainly attributed to their large consumption since they are usually prescribed for long-term use. Moreover, their poor removal in WWTPs enables their detection in the effluents along with their metabolites, conjugates or other transformation products stemming from abiotic processes, which finally end up in receiving surface waters. The latter is of great importance since many metabolites are bio-active and might occur at much higher concentrations than the parent compound [2]. Although the environmental risk caused by the concentration levels of the antidepressants in surface waters appears to be low, the likelihood of additive effects caused by exposure to mixtures of the antidepressants has been documented [2,3]. Given that antidepressants and their transformation products with similar pharmacological activity often coexist in aquatic environments, their combined presence may lead to additive or even synergistic toxic effects, resulting in an overall toxicity that can be higher than that estimated for individual compounds alone [4].

Alternative approaches to wastewater treatment have been implemented to improve environmental sustainability and overcome the shortcomings of conventional treatment methods. Advanced oxidation processes proved to be effective in the elimination of various pharmaceuticals. Among the comprising processes, the photo-induced ones are highly effective, and although they are based on the usage of various irradiation sources or lamps, they still have the advantageous potential of using solar light. Semiconductor photocatalysis has accorded great significance due to the high efficiency in mineralizing a variety of organic compounds. Titanium dioxide (TiO_2_), as a representative of photocatalytic technology, stood out owing to its high efficiency, low cost and non-toxicity. However, several obstacles limit its application on an industrial scale [5]. The catalyst is mainly used in slurries, thus requiring a post-separation step that increases the operational costs. Moreover, agglomeration of the catalyst’s particles may also occur, reducing the expected efficiency. Furthermore, TiO_2_ has a rather high bandgap energy, which implies that it requires mainly UV energy for its activation, thus limiting the usage of sunlight [6]. Moreover, when employed in slurries within a large-scale unit, TiO_2_ particles tend to disperse throughout the solution and, to some extent, precipitate at the bottom of the reactor. This phenomenon results in suboptimal light energy utilization, as only 20% of visible light and 1% of UV light can penetrate to a depth of 0.5 m underwater [7].

Aiming to overcome these issues, floating TiO_2_ is an attractive solution for increasing sunlight exploitation given the lower energy attenuation in air compared to water. Several supports working as carriers of TiO_2_ have been explored, like graphite, perlite, vermiculite, cork, or polymers [6]. Polymer-based supports are gaining popularity due to their characteristics such as high durability, hydrophobic nature, low density, mechanical strength, chemical inertness and cost-effectiveness [8]. Polystyrene, polypropylene, polyethylene or biopolymers like alginate have been applied with success, exhibiting pros and cons regarding their temperature and chemical resistance, flexibility and stability [8]. In the context of green chemistry and benefiting from lower costs, recycled poly(ethylene terephthalate) (PET) derived from plastic bottles was evaluated by our team as an efficient carrier of TiO_2_ [9]. PET was selected due to its low cost, recyclability, mechanical strength and flexibility. Moreover, its widespread use in food packaging, combined with its recyclability, urges the application of reuse practices aimed at improving sustainability.

Besides facilitating catalyst recovery, coupling TiO_2_ with magnetic Fe_3_O_4_ nanoparticles has attracted considerable research interest because it may also enhance the intrinsic photocatalytic performance of TiO_2_. Fe_3_O_4_ possesses a narrow band gap and has been reported to act synergistically with TiO_2_. Previous studies have suggested that the coupling of the two oxides may facilitate charge separation, suppress electron–hole recombination, and extend the optical response of TiO_2_ toward the visible region, thereby enhancing photocatalytic performance [10,11]. The magnetic properties of Fe_3_O_4_ constitute an additional advantage, enabling rapid and efficient catalyst recovery by applying an external magnetic field, thus improving the reusability of the photocatalyst and reducing operational costs [12]. Consequently, Fe_3_O_4_/TiO_2_ hybrid materials have emerged as promising multifunctional photocatalysts combining enhanced photocatalytic activity with facile magnetic separation, making them particularly attractive for sustainable wastewater treatment applications.

To take our research one step further, the incorporation of magnetic Fe_3_O_4_/TiO_2_ hybrid nanoparticles into floating PET-based composites was envisaged as an effective strategy to combine the merits of catalyst immobilization with magnetic separation. Beyond facilitating catalyst recovery, the Fe_3_O_4_/TiO_2_ hybrid is expected to extend the optical absorption of the photocatalyst towards the visible region and to provide additional Fe^2+^/Fe^3+^-mediated pathways for the generation of reactive species, while maintaining the buoyancy of the PET support for better sunlight utilization. In this light, the aim of our study was the synthesis and characterization of new floating magnetic PET–Fe_3_O_4_/TiO_2_ composite beads and their application in the photocatalytic degradation of a mixture of three antidepressants: venlafaxine (VNF), bupropion (BUP) and sertraline (SER). The specific pharmaceutical compounds were selected based on their high consumption, their poor removal and their toxic properties. VNF is one of the most prescribed drugs [13,14], frequently detected in surface waters [15] and in wastewater [1]. Its removal in wastewater treatment plants (WWTPs) has been proved to range from negative to low, contributing in this way to high concentrations in the effluents and ultimately the environment [1]. Moreover, ecotoxicity assessment of VNF and some of its major metabolites revealed their toxic properties to aquatic organisms [1]. Bupropion also has poor removal efficiency in WWTPs, exhibiting high concentration levels in surface waters [16,17,18], while ecotoxicity assessment of BUP and its major photolytic products showed that they range between “harmful” and “very toxic” at all trophic levels [19]. Finally, sertraline was also detected in wastewater and surface waters [20], exhibiting low to moderate removal efficiencies in WWTPs [21]. Therefore, the main objectives of this study were: (a) to synthesize three types of floating PET–Fe_3_O_4_/TiO_2_ beads with different inorganic filler loadings; (b) to characterize the prepared composites using various analytical techniques; (c) to evaluate their photocatalytic performance in the degradation of a mixture of antidepressants; (d) to assess their performance under pilot-scale continuous-flow operating conditions, simulating realistic treatment scenarios; (e) to examine their reusability; and (f) to assess the extent of mineralization achieved during the photocatalytic treatment process. Accordingly, the present work introduces a multifunctional floating magnetic photocatalyst in which recycled PET serves as a lightweight support for Fe_3_O_4_/TiO_2_ nanoparticles, combining efficient photocatalytic activity with buoyancy, facile magnetic recovery, and enhanced reusability for sustainable water treatment applications.

## 2. Materials and Methods

### 2.1. Reagents

VNF, BUP and SER (99% purity) were all purchased from Sigma Aldrich (Darmstadt, Germany). For the preparation of stock solutions of the target compounds, ultra-pure water was used, and then they were stored at −20 °C. Working solutions were prepared daily prior to photocatalytic treatment, setting 2 mg/L of each pharmaceutical as the initial concentration. For the analysis, LC-MS-grade methanol was purchased from Merck (Darmstadt, Germany), and formic acid (HCOOH) from Sigma Aldrich (Darmstadt, Germany). The leachate sample was collected from a landfill site in northern Greece, placed in amber glass vessels and stored at 4 °C to prevent further biological degradation of the sample. The initial physicochemical characteristics were: pH 7.9; total solids 21,335 mg L^−1^; COD 5840 mg L^−1^; BOD 123 mg L^−1^; NH^4+^-N 544 mg L^−1^; NO^3−^-N 295 mg L^−1^; and NO^2−^-N 998 mg L^−1^. The wastewater effluent was from an urban wastewater treatment plant (WWTP), and had the following characteristics (BOD = 24.3 mg L^−1^; COD = 95.6 mg L^−1^; SS = 12.4 mg L^−1^; and pH = 7.6).

For the synthesis of the magnetic photocatalyst, iron(III) chloride hexahydrate (FeCl_3_·6H_2_O, 98%) was purchased from Merck (Darmstadt, Germany), whereas iron(II) sulfate heptahydrate (FeSO_4_·7H_2_O) was purchased from Alfa Aesar (GmbH, Karlsruhe, Germany). Potassium hydroxide (KOH) and hydrochloric acid (HCl, 37%) were obtained from Merck (Darmstadt, Germany). Poly(ethylene glycol) (PEG 2000, with molecular weight 2000 g/mol), titanium(IV) butoxide (TBT, reagent grade, 97%) and acetylacetone (≥99%, AcAc) were supplied by Sigma-Aldrich Chemie (GmbH, Steinheim, Germany). Ethanol (analytical grade) and deionized water were used during the synthesis and purification of the magnetic nanoparticles, while high-purity nitrogen gas was employed to maintain an inert atmosphere during the co-precipitation process.

Post-consumer poly(ethylene terephthalate) (PET) bottles were used as the polymeric matrix for the preparation of the composite beads. Prior to use, the PET bottles were thoroughly rinsed with hot water and vacuum-dried at 110 °C for 12 h. Trifluoroacetic acid (TFA, ≥99%), chloroform (CHCl_3_, anhydrous, ≥99%) and acetone (analytical grade) were purchased from Sigma Aldrich chemical Co. (Darmstadt, Germany).

### 2.2. Synthetic Pathways

#### 2.2.1. Synthesis of Hybrid Fe_3_O_4_/TiO_2_ Particles

For the synthesis of magnetic Fe_3_O_4_/TiO_2_ particles, a co-precipitation method was employed [22]. Briefly, 3.375 g (12.5 mmol) of FeCl_3_·6H_2_O and 1.738 g (6.25 mmol) of FeSO_4_·7H_2_O, corresponding to a Fe^3+^:Fe^2+^ molar ratio of 2:1, were separately dissolved in 100 mL of deionized water. The two solutions were then thoroughly mixed, and 50 mL of a 2 M KOH aqueous solution was rapidly added to the stirred mixture to initiate precipitation according to the Fe^2+^ + 2Fe^3+^ + 8OH^−^ → Fe_3_O_4_ + 4H_2_O [22,23]. The suspension was maintained under continuous stirring at 80 °C for 1 h under a nitrogen atmosphere. While the suspension was still held at 80 °C, PEG2000 (5 mL, aqueous solution) was added dropwise over the final period of stirring, acting as a surfactant to prevent particle agglomeration and to promote uniform dispersion of magnetic nanoparticles. Magnetite (Fe_3_O_4_) was formed as a black precipitate, which was washed twice with ethanol and deionized water and subsequently separated using a neodymium magnet. The final product was left at room temperature to allow solvent evaporation and was then completely dried in an oven at 60 °C for 1 h.

Fe_3_O_4_/TiO_2_ hybrid magnetic nanoparticles, consisting of magnetic Fe_3_O_4_ and TiO_2_ particles, were synthesized via a sol–gel suspension method. For this purpose, the as-prepared Fe_3_O_4_ nanoparticles were dispersed in distilled water in a three-neck round-bottom flask equipped with a reflux condenser, and a 0.1 mol L^−1^ HCl solution was added. The dispersion was ultrasonically treated. Separately, 15.9 g (45.3 mmol) of TBT was pre-treated with acetylacetone at a molar ratio of AcAc:Ti = 0.2:1 to form a chelated, less hydrolyzable Ti complex [24], and this pre-chelated TBT was then added gradually to the acidified Fe_3_O_4_ dispersion under continuous stirring. The mixture was stirred at 50 °C for approximately 2 h to form a colloidal dispersion, allowing hydrolysis–condensation of the chelated titanium precursor onto the Fe_3_O_4_ surface. The resulting suspension was further stirred for 4 h to promote sol–gel condensation and the formation of TiO_2_ in intimate contact with Fe_3_O_4_ nanoparticles [24,25].

#### 2.2.2. Preparation of Composite PET-Fe_3_O_4_/TiO_2_ Beads

Composite PET–Fe_3_O_4_/TiO_2_ beads were prepared using a non-solvent precipitation technique, as also followed in our previous work [9]. Initially, recycled PET (1 g) was dissolved in the minimum amount of a CHCl_3_/TFA solvent mixture (85/15% *v*/*v*) to obtain a viscous polymer solution. Subsequently, the appropriate amount of Fe_3_O_4_/TiO_2_ nanoparticles was added to the polymer solution to obtain final inorganic loadings of 10, 30, and 50 wt% relative to the polymer mass. The resulting suspension was sonicated using an ultrasonic homogenizer (UP-400S, Hielscher Ultrasonics GmbH, Teltow, Germany) for 5 min to ensure homogeneous dispersion of the nanoparticles within the polymer matrix. The nanocomposite suspension was then added dropwise through a syringe equipped with a fine needle into a non-solvent for PET, inducing phase inversion and precipitation of the polymer in the form of spherical beads. The resulting composite particles were collected from the precipitation medium, air-dried overnight at room temperature, and subsequently dried in an oven at 60 °C for 1 h to ensure complete solvent removal.

The prepared PET–Fe_3_O_4_/TiO_2_ beads combined buoyancy with magnetic separability, facilitating their recovery from the treated aqueous medium and enhancing their potential for repeated use in photocatalytic applications. Magnetic recoverability of the PET–Fe_3_O_4_/TiO_2_ composite beads was demonstrated visually, as shown in Supplementary Videos S1–S4. The morphology, surface characteristics, shape, and particle size of the synthesized beads were initially examined using a stereomicroscope (Zeiss SteREO Discovery V.20, Carl Zeiss Microscopy GmbH, Jena, Germany) equipped with a high-resolution camera (ProgRes GRYPHAX Altair, JENOPTIK Optical Systems GmbH, Jena, Germany). The bead diameter was determined from the stereomicroscope images using the GRYPHAX image analysis software. The beads were nearly spherical, with an average diameter of 1.92 ± 0.20 mm (*n* = 5). The obtained materials were further characterized with respect to their physicochemical properties using the analytical techniques described below.

### 2.3. Characterization of Synthesized Materials and Composite Beads

Fourier transform infrared (FTIR) spectra of the synthesized PET–Fe_3_O_4_/TiO_2_ composite beads were recorded using a PerkinElmer Spectrum One FTIR spectrometer (PerkinElmer, Dresden, Germany) employing KBr pellets. Spectra were collected in absorbance mode over the wavenumber range of 4000–450 cm^−1^ with a spectral resolution of 4 cm^−1^ and 32 accumulated scans. The FTIR spectra of the composite beads recovered after the second photocatalytic cycle were recorded in attenuated total reflectance (ATR) mode.

The thermal stability of the synthesized beads was evaluated by thermogravimetric analysis (TGA) using a NETZSCH STA 449 F5 thermal analyzer (NETZSCH, Selb, Germany). Samples were heated from 30 to 600 °C at a heating rate of 20 °C min^−1^ under a nitrogen atmosphere (99.9%) with a flow rate of 30 cm^3^ min^−1^.

The morphology and elemental composition of the Fe_3_O_4_/TiO_2_ particles and the prepared PET–Fe_3_O_4_/TiO_2_ composite beads were investigated using a JEOL JSM-7610F field-emission scanning electron microscope (JEOL Ltd., Tokyo, Japan) equipped with an Oxford AZtec Energy Advanced X-act energy-dispersive X-ray spectroscopy (EDS) system (Oxford Instruments NanoAnalysis, High Wycombe, UK). Cross-sectional specimens were coated with carbon to ensure adequate electrical conductivity during analysis. SEM–EDS measurements were performed at accelerating voltages of 5–20 kV, a probe current of 45 nA, and a counting time of 60 s.

X-ray diffraction (XRD) patterns of the studied samples were recorded using a Rigaku Miniflex 600 diffractometer (Rigaku Corporation, Tokyo, Japan) employing Cu Kα radiation (λ = 1.5406 Å) for phase identification. Diffractograms were collected over the 2θ range of 10–80°.

For the prepared photocatalyst, a nitrogen adsorption/desorption experiment at −196 °C was performed on an Automatic Volumetric Sorption Analyzer (Autosorb-1 MP, Quantachrome Instruments, Boynton Beach, FL, USA). The samples were previously outgassed at 150 °C for 16 h under 5 × 10^−9^ Torr vacuum. The BET area (total surface area) of the catalysts was determined by the multi-point BET method, the mesopore size distribution and average size by the BJH analysis of adsorption data, and the micropore volume and area by the t-plot method.

Ultraviolet–visible diffuse reflectance spectroscopy (UV–Vis DRS) in the solid state was also performed employing a JASCO V-750 spectrophotometer (JASCO Corporation, Tokyo, Japan) equipped with an INS-470 integrating sphere attachment at ambient temperature. The samples’ band gap energy was calculated using the Kubelka–Munk equation.

Differential scanning calorimetry (DSC) was employed to study the thermal transitions, i.e., crystallization/melting and glass transition of PET samples. The measurements were performed by means of a DSC TA 25 (TA Instruments, New Castle, DE, USA) in a pure nitrogen atmosphere (50 mL/min), in the temperature range from 10 to 295 °C, on samples of 2.5–3.0 mg in mass, where the instrument was calibrated using zinc standards. The semicrystalline polyester samples were heated at 20 °C/min up to T = T_m_ + 40 °C in order to evaluate their melting temperature (T_m_) and crystallinity. Following a holding step for 5 min, amorphous materials were obtained by implementing a cooling step at the highest rate possible in the DSC. Thus, the glass transition temperature (T_g_), cold crystallization temperature (T_cc_) and T_m_ were measured for the quenched samples during the heating step at 20 °C/min. Moreover, a slow cooling step was performed (10 °C/min) prior to melting (holding for 5 min at T_m_ + 40 °C) for the determination of the crystallization temperature (T_c_) to a temperature below T_g_ (T_g_ − 70 °C). Finally, a subsequent heating scan was conducted (20 °C/min). For each thermal transition step, crystallinity was expressed as a crystalline fraction (CF) using the following equation [26]:

(1) $$\mathrm{CF}\;(\%)\;=\;\left(\frac{{\Delta H}_{m}}{{\Delta H}_{m}^{0}*(1-\frac{\mathrm{wt}\%\;\mathrm{NPs}}{100})}\right)\times 100$$

where ∆H_m_ and ∆H°_m_ are the experimental melting enthalpy and the theoretical heat of fusion of a 100% crystalline polymer, ∆H^o^_m_ = 135 J/g for PET [27,28].

### 2.4. Photocatalytic Experiments

#### 2.4.1. Lab-Scale Experimental Setup

Preliminary photocatalytic degradation experiments of the antidepressant mixture were conducted in an Atlas Suntest CPS+ photoreactor (Atlas Material Testing Technology GmbH, Linsengericht, Germany) equipped with a 1500 W xenon lamp operating at an irradiation intensity of 500 W m^−2^. A glass filter was applied to eliminate wavelengths below 290 nm, simulating solar irradiation conditions. The aqueous solution containing the target antidepressants (initial concentration: 2 mg L^−1^ for each compound) was introduced into the reactor together with the appropriate amount of floating catalyst. Prior to irradiation, the suspension was magnetically stirred in the dark for 30 min to establish adsorption–desorption equilibrium between the pharmaceuticals and the catalyst surface. At the end of this dark period, the concentration of each antidepressant was determined and used as the initial concentration (C_0_) for the subsequent photocatalytic experiments. Thus, the reported photocatalytic degradation percentages and kinetic calculations were based on the concentrations remaining after the adsorption–desorption equilibration step. Subsequently, the light source was switched on to initiate the photocatalytic process. The solution pH was intentionally left unadjusted to evaluate the photocatalytic performance of the PET–Fe_3_O_4_/TiO_2_ floating beads under conditions that do not require chemical pH adjustment, which is desirable for practical large-scale water treatment applications.

#### 2.4.2. Pilot-Scale Setup

Experiments were also conducted in a pilot-scale photocatalytic unit consisting of the following main components: a 12 L annular photoreactor equipped with four 40 W ultraviolet (UV) lamps providing an irradiation intensity of approximately 160 W m^−2^, arranged to ensure uniform irradiation of the reaction medium; a 100 L feed/holding tank; a mechanical stirring system (rotor) providing continuous agitation of the liquid phase within the tank and operating at adjustable rotational speeds in the range of 0–100 rpm; and a centrifugal recirculation pump responsible for maintaining continuous flow of the working solution through the system, with a maximum flow capacity of 28 L/min. All components are integrated into a closed-loop recirculating hydraulic circuit, enabling continuous circulation of the aqueous solution between the reservoir tank and the photoreactor. This configuration ensures homogeneous mixing, stable hydrodynamic conditions, and consistent exposure of the reaction medium to UV irradiation throughout the photocatalytic experiments. Samples were collected at predetermined time intervals, filtered, and analyzed accordingly.

The degradation kinetics of the investigated compounds were evaluated using the simplified Langmuir–Hinshelwood model (Equation (2)), which is widely applied in heterogeneous photocatalytic studies. Under the experimental conditions employed, the degradation process was found to follow pseudo-first-order kinetics, in agreement with previous reports [9].

(2) ln(C/C_0_) = −*kt*

where *k* represents the apparent first-order rate constant, *C*_0_ is the initial concentration of the target compound, and *C* is the concentration at time *t*.

### 2.5. Analytical Processes

#### 2.5.1. Determination of Pharmaceutical Concentration

The determination of the target pharmaceutical compounds throughout the photocatalytic experiments was performed using a high-resolution LC–Q Exactive Focus Orbitrap mass spectrometer (Thermo Fisher Scientific, Waltham, MA, USA). The system was coupled with a heated electrospray ionization source (HESI-II) and consisted of a high-pressure binary solvent delivery system, an autosampler, and a temperature-controlled column compartment. Chromatographic separation was achieved on a Thermo Hypersil GOLD aQ C18 column (50 × 2.1 mm, 1.9 μm particle size). The mobile phase was composed of LC-MS-grade water containing 0.1% formic acid (solvent A) and methanol with 0.1% formic acid (solvent B). The gradient elution program was as follows: initial conditions of 10% B were maintained for 1.5 min, followed by a gradual increase to 60% B at 4 min, 70% B at 8 min, and 100% B at 11 min. The column was kept under these conditions until 12 min, after which the mobile phase composition was returned to the initial conditions (10% B) at 13 min and maintained until the end of the run (15 min). Mass spectrometric analysis was conducted in positive electrospray ionization mode. Full-scan data acquisition was performed over an m/z range of 50–1000 with a resolving power of 70,000. Fragmentation experiments were carried out using stepped normalized collision energies of 20, 30, and 45 eV. The analytical method was evaluated in terms of linearity, limit of detection (LOD), and limit of quantification (LOQ). Calibration curves were constructed over the concentration range of 1–500 μg/L for the three target pharmaceutical compounds, showing good linearity with correlation coefficients (R^2^) > 0.99. The LOQ was established at 1 μg/L, while the LOD was estimated at 0.33 μg/L.

#### 2.5.2. Mineralization Studies

In order to estimate the extent of mineralization of the studied pharmaceuticals, TOC measurements were carried out employing a Shimadzu TOC-L analyzer (Shimadzu Corporation, Kyoto, Japan) with a non-dispersive infrared (NDIR) detector.

#### 2.5.3. Analysis of Iron Leaching

For the determination of the possible leaching of iron from the synthesized beads during treatment, inductively coupled plasma mass spectrometry (ICP-MS) (model Thermo Scientific, Fisher Scientific, Ottawa, ON, Canada) with Qtegra™ Intelligent Scientific Data Solution™ Software was employed. Further analytical details can be found elsewhere.

## 3. Results and Discussion

### 3.1. Characterization of Prepared Composite PET–Fe_3_O_4_/TiO_2_ Beads

Driven by the increasing demand for sustainable technologies capable of mitigating pharmaceutical pollution in aquatic environments, considerable research efforts have focused on the development of new and eco-friendly photocatalytic materials [29,30]. Nevertheless, the immobilization of high amounts of inorganic photocatalysts into polymeric matrices without compromising their structural integrity and stability remains a tough and challenging task [31,32]. Therefore, the prepared PET–Fe_3_O_4_/TiO_2_ composite beads were herein comprehensively characterized to verify the successful incorporation of the photocatalyst particles and to assess their physicochemical properties before photocatalytic application.

**Figure 1:**
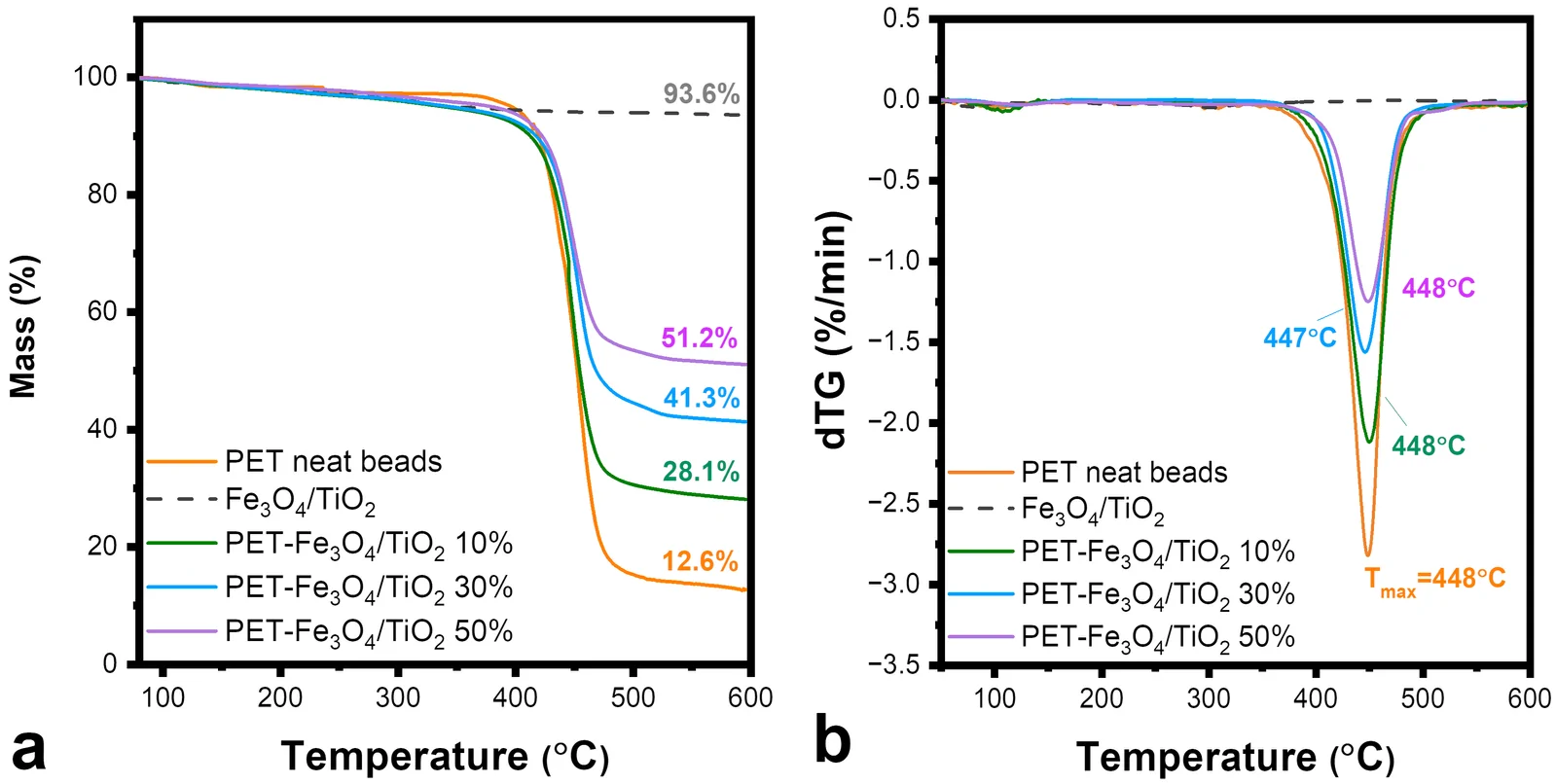
(**a**) Mass loss and (**b**) dTG curves of neat PET and the prepared composite beads (PET-Fe_3_O_4_/TiO_2_) incorporating different Fe_3_O_4_/TiO_2_ contents (10, 30, 50 wt%).

Within this framework, TGA was initially employed to evaluate the thermal stability of the synthesized PET–Fe_3_O_4_/TiO_2_ composite beads and to verify the successful immobilization of the magnetic photocatalyst within the polymeric matrix, employing three different compositions of Fe_3_O_4_/TiO_2_ filler (10–50 wt%). Specifically, the TGA and dTG thermograms are presented in Figure 1a and b, respectively. Neat recycled PET exhibited excellent thermal stability, with the onset of thermal degradation occurring above 360 °C and a maximum degradation rate (T_max_) at approximately 448 °C. Similar to neat PET, all composite beads displayed a single major degradation step within the 350–510 °C temperature range, corresponding to the thermal decomposition of the polymer matrix. The incorporation of Fe_3_O_4_/TiO_2_ nanoparticles had only a marginal effect on the thermal degradation behavior of PET, as the maximum degradation temperatures remained essentially unchanged, exhibiting only a slight shift towards lower values (approximately 1–2 °C). This negligible variation indicates that the addition of the magnetic photocatalyst does not adversely affect the thermal stability of the polymer matrix, while preserving the characteristic degradation profile of PET.

The residual mass at 600 °C increased progressively with increasing Fe_3_O_4_/TiO_2_ loading, from 12.6% for neat PET to approximately 28.1%, 41.3% and 51.2 wt% for the PET–Fe_3_O_4_/TiO_2_ 10 wt%, PET–Fe_3_O_4_/TiO_2_ 30 wt%, and PET–Fe_3_O_4_/TiO_2_ 50 wt% beads, respectively. The abovementioned trend is attributed to the non-degradable nature of the inorganic Fe_3_O_4_/TiO_2_ nanoparticles, which remain almost thermally stable under the applied experimental conditions. Considering that the synthesized Fe_3_O_4_/TiO_2_ nanoparticles exhibited a residual mass of 93.6 wt% at 600 °C, the experimentally determined char values of the composite beads were in good agreement with the nominal inorganic contents, confirming the successful immobilization and retention of the magnetic photocatalyst within the PET matrix.

**Figure 2:**
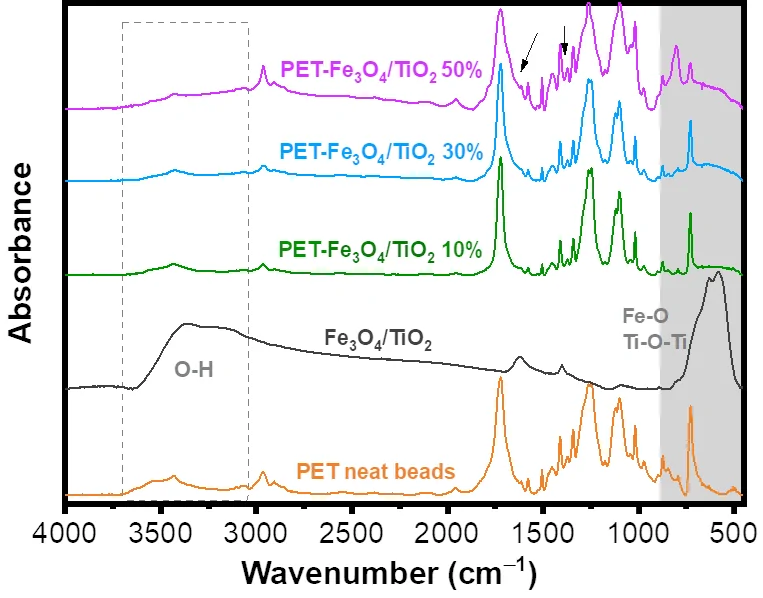
FTIR spectra of neat PET beads, Fe_3_O_4_/TiO_2_ particles and PET–Fe_3_O_4_/TiO_2_ composite beads.

FTIR spectroscopy was further employed to investigate the chemical structure of the synthesized materials and to assess possible interactions between the PET matrix and the Fe_3_O_4_/TiO_2_ nanoparticles (Figure 2). The FTIR spectrum of the Fe_3_O_4_/TiO_2_ nanoparticles exhibited broad absorption bands around 3400 and 1630 cm^−1^, which are attributed to stretching and bending vibrations of surface hydroxyl groups and adsorbed water molecules, respectively. In addition, the broad absorption region between 450 and 800 cm^−1^ corresponds to overlapping Ti–O and Fe–O vibrational modes of TiO_2_ and Fe_3_O_4_, respectively [33,34].

The spectrum of neat PET displayed the characteristic absorption bands of the polyester structure, including the intense carbonyl stretching vibration at approximately 1720 cm^−1^, the aromatic C=C stretching vibrations in the 1600–1500 cm^−1^ region, the C–O–C stretching vibrations of the ester groups between 1240 and 1100 cm^−1^, and the out-of-plane C–H bending vibrations below 900 cm^−1^ [35]. These characteristic PET bands remained essentially unchanged after the incorporation of Fe_3_O_4_/TiO_2_ nanoparticles, indicating that the immobilization process did not alter the chemical structure of the polymer matrix.

Nevertheless, a gradual increase in the absorption intensity within the 450–800 cm^−1^ region was observed with increasing Fe_3_O_4_/TiO_2_ loading, reflecting the progressively higher contribution of the inorganic phase. A slight broadening of the carbonyl band around 1720 cm^−1^ was also observed for the composite beads, which may be associated with weak interfacial interactions between the hydroxylated surface of the Fe_3_O_4_/TiO_2_ nanoparticles and the PET matrix [9]. However, the absence of any significant band shifts or the appearance of new absorption bands suggests that no new covalent bonds were formed during composite preparation, indicating that the nanoparticles are predominantly physically immobilized within the PET matrix. These findings are consistent with the successful incorporation of the magnetic photocatalyst while preserving the structural integrity of the recycled PET support.

**Figure 3:**
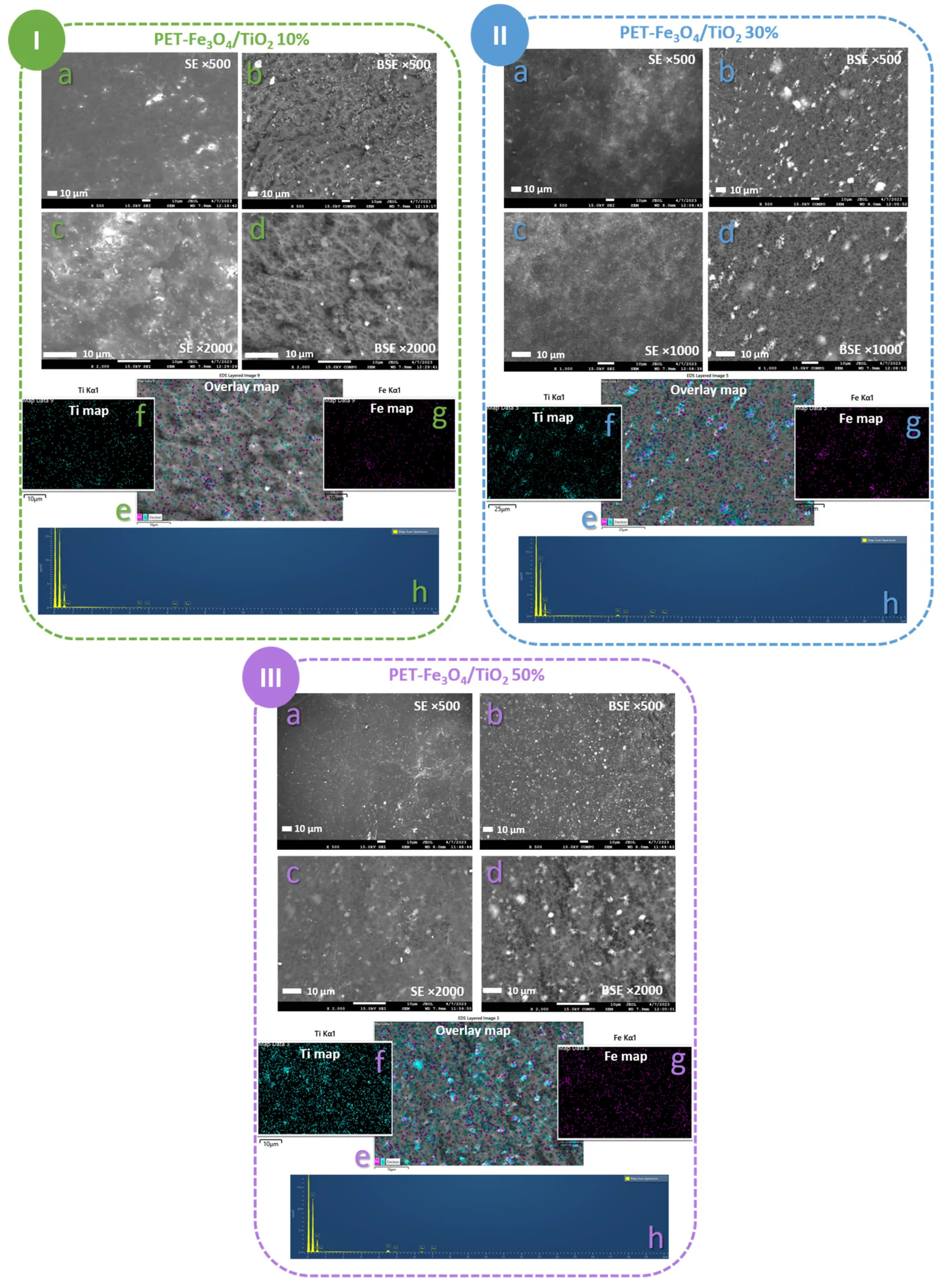
SEM–EDS characterization of the PET–Fe_3_O_4_/TiO_2_ composite beads with (**I**) 10 wt%, (**II**) 30 wt% and (**III**) 50 wt% Fe_3_O_4_/TiO_2_ content. For each composition, the panels show: (**a**) secondary electron (SE) and (**b**) backscattered electron (BSE) images at ×500; (**c**) SE and (**d**) BSE images at higher magnification (×2000 for I and III; ×1000 for II); (**e**) overlay EDS elemental map; individual elemental maps of (**f**) Ti (cyan) and (**g**) Fe (magenta); and (**h**) the corresponding EDS map sum spectrum.

Aiming to acquire information about the morphology and elemental distribution of the prepared PET–Fe_3_O_4_/TiO_2_ composite beads, SEM/EDS was utilized. SEM micrographs, along with the related images employing the backscattered electron detector, as depicted in Figure 3, demonstrated a satisfactory dispersion of the Fe_3_O_4_/TiO_2_ nanoparticles throughout both the surface and cross-sectional regions of the beads, with no evidence of extensive particle agglomeration. The bead surfaces appeared generally compact and relatively smooth (Figure 3I,II), although localized wrinkles and microcracks were observed, particularly at higher nanoparticle loadings, as in the sample PET–Fe_3_O_4_/TiO_2_ 50wt% (Figure 3III). Such morphological features are commonly associated with solvent evaporation during bead formation and with internal stresses generated during phase inversion and drying, as well as the higher contents of inorganic filler, creating irregularities throughout bead formation [36]. Elemental mapping confirmed the homogeneous dispersion of both Ti and Fe throughout the polymer matrix, providing strong evidence for the successful immobilization of the magnetic photocatalytic particles. The absence of large particle-rich domains further suggests efficient distribution of the inorganic phase within the PET support, indicating that PET could successfully facilitate the immobilization of Fe_3_O_4_/TiO_2_ contents up to 50 wt%. The morphology and elemental distribution of the prepared hybrid Fe_3_O_4_/TiO_2_ particles were also examined by SEM–EDS (please see Supplementary Material Figure S3). The particles consisted of aggregated nanosized primary particles forming a porous network (Figure S3a,b). Elemental mapping (Figure S3d–g) showed that Ti was distributed throughout the sample, whereas Fe was mainly concentrated in discrete domains of a few micrometers dispersed within the Ti-rich matrix.

**Figure 4:**
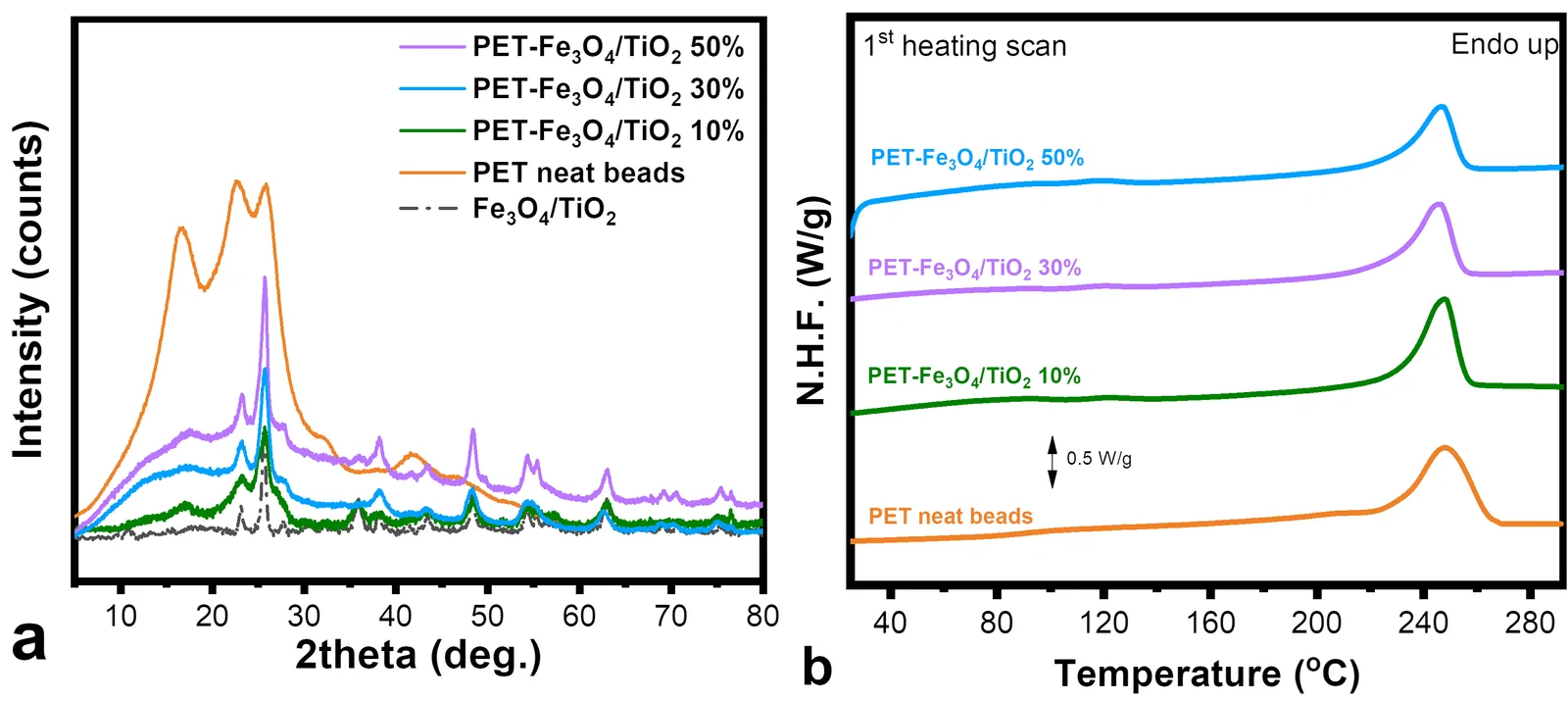
(**a**) XRD patterns and (**b**) DSC thermographs (first heating scan) of the neat PET and composite PET–Fe_3_O_4_/TiO_2_ beads (N.H.F. stands for normalized heat flow).

The results discussed above could also be supplemented by the XRD data, with the XRD patterns of neat PET, Fe_3_O_4_/TiO_2_ nanoparticles, and PET–Fe_3_O_4_/TiO_2_ composite beads being presented in Figure 4a. The diffraction pattern of neat PET exhibited the characteristic reflections of a semicrystalline polyester, with the most prominent diffraction peaks located at approximately 16.6°, 23.2° and 25.6° across the pattern’s hump, in agreement with previous reports for PET [37]. As far as the Fe_3_O_4_/TiO_2_ nanoparticles’ diffractograms are concerned, the reflections at 25.3°, 38.1°, 48.4°, 54.2°, 63.1°, and 75.3° can be assigned to the anatase phase of TiO_2_, corresponding to the (101), (004), (200), (105), (204), and (215) crystallographic planes, respectively, whereas the diffraction peak at 27.6° is characteristic of rutile TiO_2_ and corresponds to the (110) plane. These findings indicate that the TiO_2_ phase consists of a mixed anatase/rutile structure, which has frequently been associated with enhanced photocatalytic performance due to improved charge separation at the phase interface [32,38,39,40]. Similar diffraction features have been reported for Fe_3_O_4_/TiO_2_ hybrid photocatalysts prepared via sol–gel routes, as reported in the literature [34,38,41]. The diffraction peak observed at approximately 43.5° is attributed to the spinel structure of magnetite (Fe_3_O_4_), confirming the preservation of the magnetic phase after the deposition of TiO_2_. Although some characteristic Fe_3_O_4_ reflections exhibit relatively low intensity, this behavior is commonly observed in Fe_3_O_4_/TiO_2_ hybrid nanoparticles and can be attributed to the lower magnetite content, peak overlap with TiO_2_ reflections, and the shielding effect exerted by the surrounding matrix. Similar observations have been reported for TiO_2_/Fe_3_O_4_ systems in which the diffraction peaks of the magnetic phase become partially masked by the dominant TiO_2_ reflections [33]. Moreover, no reflections attributable to hematite (α-Fe_2_O_3_), such as the characteristic (104) reflection at ~33.2°, were detected, indicating that no significant oxidation of the magnetic phase to hematite occurred; nevertheless, a partial surface oxidation of magnetite to maghemite (γ-Fe_2_O_3_) cannot be excluded on the basis of XRD alone, since the two phases exhibit very similar diffraction patterns.

Concerning the PET–Fe_3_O_4_/TiO_2_ composite beads, the characteristic diffraction peaks of the Fe_3_O_4_/TiO_2_ nanoparticles remained detectable in all tested samples, confirming the successful incorporation of the magnetic photocatalyst into the polymer matrix. Furthermore, a progressive decrease in the intensity of the PET crystalline reflections was observed with increasing nanoparticle loading. This reduction could be attributed to the presence of the Fe_3_O_4_/TiO_2_ nanoparticles, which hinder the regular packing and crystallization of PET macromolecular chains by restricting chain mobility and crystal growth [42]. The simultaneous presence of PET, TiO_2_, and Fe_3_O_4_ diffraction features therefore provides strong evidence for the successful formation of PET–Fe_3_O_4_/TiO_2_ composite beads without significant alteration of the crystalline structure of the embedded magnetic photocatalyst.

The textural and optical properties of the hybrid Fe_3_O_4_/TiO_2_ particles were further evaluated by N_2_ porosimetry and UV–Vis DRS (Figure S2). The particles exhibited a type IV isotherm with an H3 hysteresis loop at P/P_0_ > 0.85 (Figure S2a), characteristic of mesoporous aggregates of nanoparticles with slit-shaped interparticle voids. The BET specific surface area was 65 m^2^ g^−1^ and the total pore volume was 0.519 cm^3^ g^−1^ (at P/P_0_ = 0.99), while the t-plot analysis revealed no microporosity, indicating that the porosity arises from the voids between the aggregated primary particles. The UV–Vis DRS spectrum (Figure S2b) showed low reflectance (~10%) over the entire UV and visible region up to ~500 nm, reflecting the strong light absorption of the iron oxide phase, while the reflectance increased markedly above ~550 nm. The absorption edge estimated from the Tauc plot using the Kubelka–Munk function was ~1.9 eV (~650 nm) (Figure S2c), in good agreement with the value of 1.88 eV reported for sol–gel-derived TiO_2_–Fe_3_O_4_ immobilized in alginate beads and close to that of bare Fe_3_O_4_ (1.68 eV) [33,34]. This indicates that the optical response of the hybrid particles is dominated by the iron oxide phase, which extends the absorption over the visible region. The intrinsic absorption edge of anatase TiO_2_ (~3.2 eV) could not be clearly resolved, as it is masked by the intense absorption of the iron oxide component, while the presence of crystalline anatase was confirmed by XRD (Figure 4a).

Moreover, DSC was employed to investigate the influence of Fe_3_O_4_/TiO_2_ incorporation on the thermal transitions and crystalline structure of the recycled PET matrix; the corresponding thermograms are presented in Figure 4b as normalized heat flow (N.H.F.) versus temperature. As summarized in Table 1, neat PET beads exhibited a melting temperature (T_m_) of 247.9 °C and a crystalline fraction (CF) of 32.5% [37]. The incorporation of Fe_3_O_4_/TiO_2_ nanoparticles resulted in a slight decrease in the melting temperature, with T_m_ varying between 247.8 and 246.6 °C, indicating that the presence of the inorganic phase had only a minor influence on the melting behavior of PET [43].

**Figure 5:**
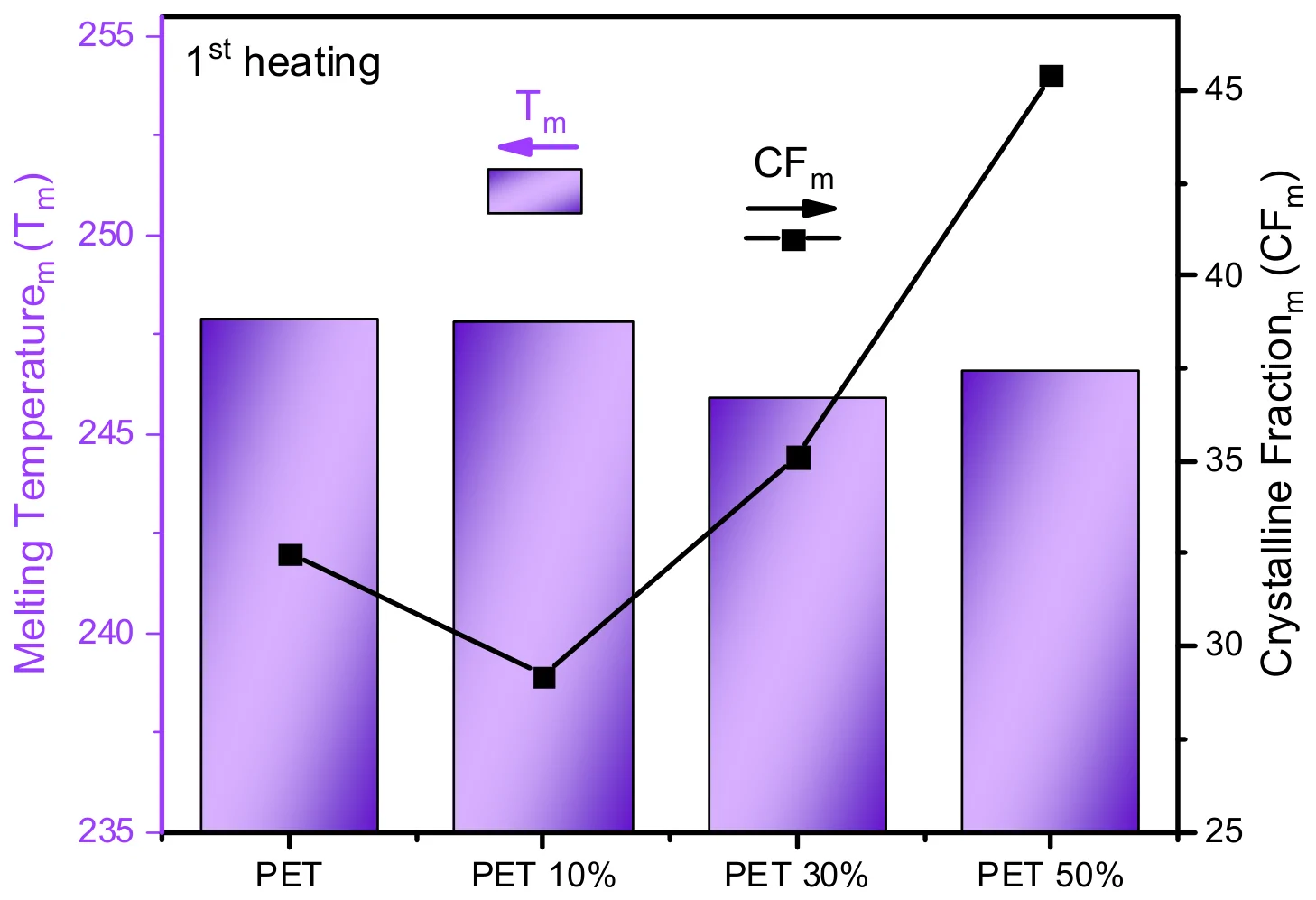
Melting peak and crystalline fraction (CF_m_) during the first heating from the glassy state.

Regarding the calculated crystalline fraction (CF_m_) at the melting temperature, it decreased from 32.5% for neat PET to 29.2% for the composites containing 10 wt% Fe_3_O_4_/TiO_2_ (Figure 5). This reduction suggested that the dispersed nanoparticles interfere with the regular packing of PET macromolecular chains, restricting crystal growth and reducing the overall crystalline fraction. Interestingly, the composites containing 30 wt% and 50 wt% Fe_3_O_4_/TiO_2_ exhibited higher crystallinity (35.1% and 45.4%, respectively) compared with the neat PET beads and minor compositions. This behavior may indicate that, at sufficiently high filler loading, the Fe_3_O_4_/TiO_2_ particles partially acted as heterogeneous nucleating sites, promoting PET crystallization. Similar competing effects between crystal growth inhibition and heterogeneous nucleation have been reported for highly filled polymer nanocomposites [44]. However, previous work by our group has shown that the PET composite beads based on TiO_2_ NPs did not follow a similar trend, as the crystallinity at high loadings (30 wt%) decreased. This may imply that the iron oxides in the present work have been embedded well together with the titania in the PET matrix and have promoted the development of additional nucleation sites [9]. All the thermal values calculated by DSC and TGA are summarized in Table 1.

**Figure 6:**
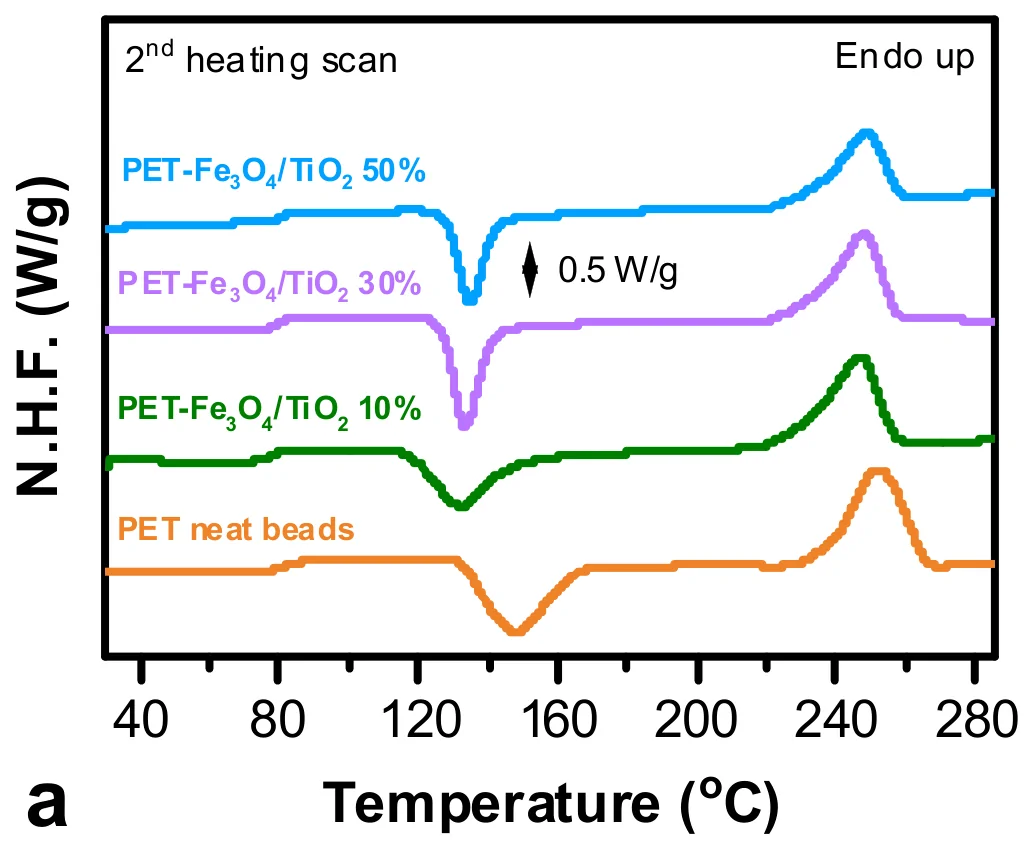

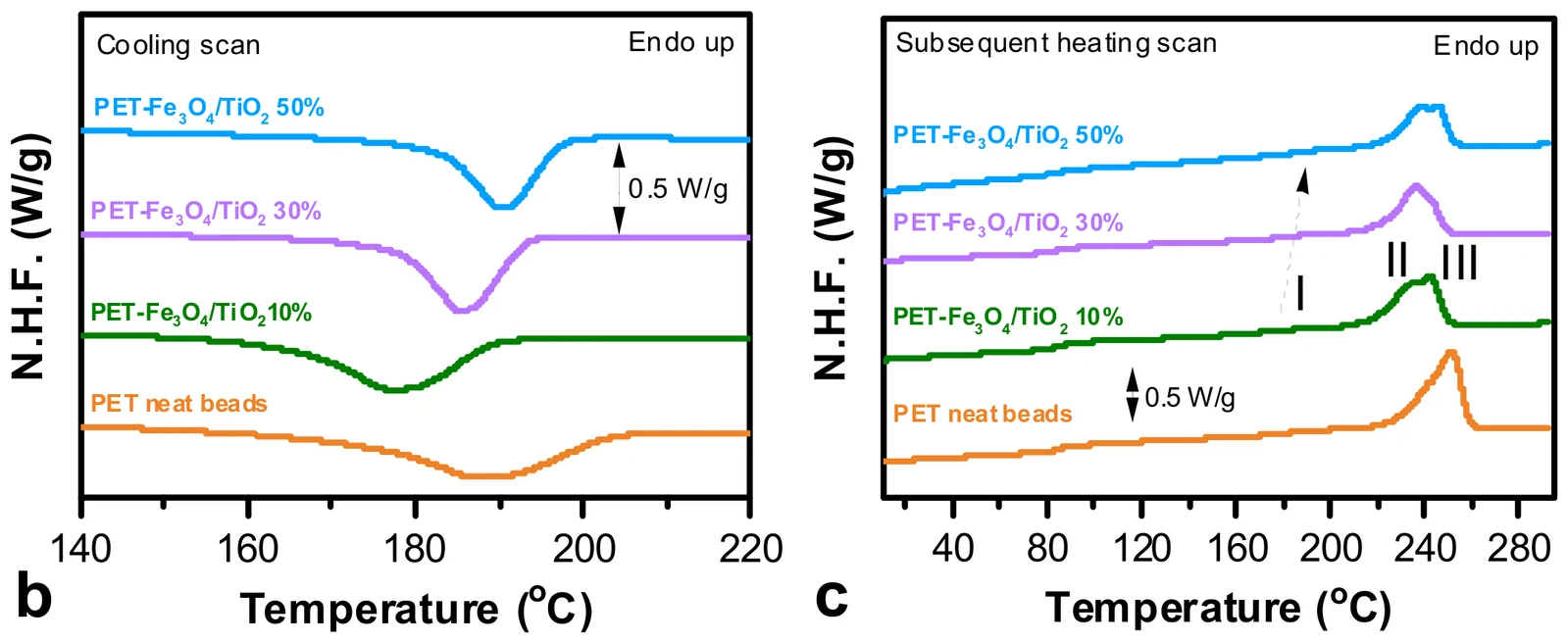
DSC traces during (**a**) the second heating (quenched samples) (20 °C/min), (**b**) slow crystallization (cooling scan—10 °C/min) and (**c**) the subsequent heating (20 °C/min) of melt-crystallized samples. In panel (c), peaks I, II, and III correspond to the melting of less-perfect crystals, primary crystals, and crystals formed through recrystallization or reorganization during heating, respectively.

The second heating scan (Figure 6a) for the quenched samples of PET composites was also recorded. The glass-transition temperature (T_g_) exhibited only a minor variation with respect to the NP content. In the samples containing 10 and 30 wt% NPs, T_g_ decreased by 2 °C, suggesting a slight increase in the chain mobility of PET macromolecular chains. This probably happened due to the enhanced interaction between the NPs and the rigid amorphous fraction (RAF) of PET chains, creating the necessary space between the macromolecular chains by increasing the free volume, thus improving the chain mobility. This may also be facilitated by the sufficient mixing method that was used in the current work. However, at 50 wt% NPs, T_g_ increased but did not exceed that of the neat PET beads. The latter may have occurred because of the restricted chain mobility caused by the significantly higher crystallinity of the composite samples (Figure 5) [45]. The PET/TiO_2_ beads reported by Evgenidou et al. exhibited a completely different behavior, as the Tg was increased for all the bead samples [9]. This may be attributed to the presence of iron oxide in the Fe_3_O_4_/TiO_2_ hybrid material, which may have promoted more effective dispersion of the NPs throughout the PET matrix and prevented their agglomeration between the PET macromolecular chains, even at the high loading (50 wt%, Table 2) [9].

Figure 6a also shows the variation in cold crystallization (T_cc_) and T_m_ of the examined materials. Neat PET exhibited a relatively broad T_cc_ exothermic peak at a high temperature (147.9 °C) (Table 2). Notably, with increasing NP content, the T_cc_ progressively decreased (132.9–134.9 °C), while the exothermic peak became considerably sharper. The reduction of T_cc_ indicated that the NPs promoted the crystallization of the initially amorphous PET composite samples during heating. This behavior can be attributed to the heterogeneous nucleation effect of the NPs, which provided numerous sites for the formation of PET crystal nuclei, whereas this trend can also be correlated with the slightly increased chain mobility observed through the reduction of T_g_ (Table 2) [44].

Furthermore, regarding melt crystallization, Figure 6b shows the cooling traces of the materials during crystallization from the melt. Neat PET exhibited a broad crystallization exothermic peak (T_c_) at 188 °C, while with increasing NP content, T_c_ shifted to lower temperatures, displaying sharper exothermic peaks (for 30 and 50 wt% NP content). In contrast, the sample with the highest content of NPs exhibited the sharpest T_c_ among the samples at even higher temperatures; specifically, this reflected a nucleation phenomenon dominated by the large number of nanoparticle surfaces, which provided a plethora of nucleation sites, promoting faster crystallization and uniform crystal growth of the PET macromolecular polymer chains. This non-linear variation regarding the trend of T_c_ and sharpness (Figure 6b) may indicate different crystallization rates or morphological changes under crystallization from the melt, which requires the implementation of in-depth analysis of melt crystallization kinetics [46].

**Figure 7:**
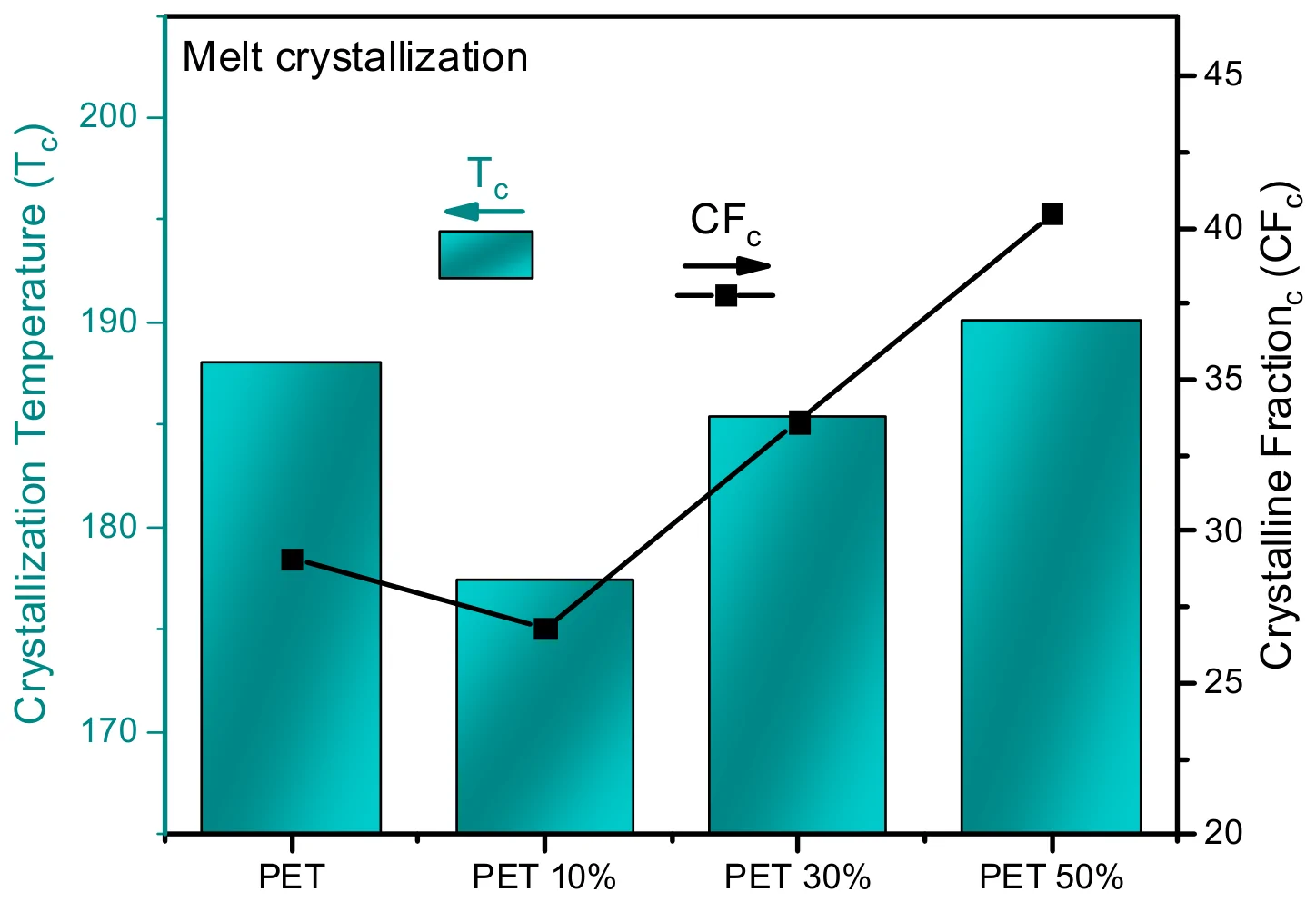
Crystallization peak and crystalline fraction (CF_c_) during slow crystallization from the melt.

The crystalline fraction during cooling (CF_c_, Figure 7) followed the same trend, increasing progressively along with the NP content and reached a maximum value of 40% for the PET 50 wt% sample. The latter had a significant impact on the first melting peak (I) during the subsequent heating step (Figure 6c). In this case, it shifted by approximately 10 °C to higher temperatures, which resulted in crystals with higher thermodynamic stability, compared to those in the samples containing 10 and 30 wt% NPs. The multiple melting behavior was attributed to the presence of crystal populations with different degrees of perfection: peak I was associated with the melting of defective or less-perfect crystals, peak II with the melting of the primary crystals, and peak III with the melting of crystals formed through recrystallization or crystal reorganization during heating [47].

Beyond their structural and thermal characteristics, the successful incorporation of the Fe_3_O_4_/TiO_2_ phase into the PET matrix did not compromise the buoyancy of the composite beads, which remained afloat throughout preparation and subsequent use. In contrast to the neat PET beads, which showed no response to an external magnetic field (Video S1), the Fe_3_O_4_/TiO_2_-loaded beads exhibited strong magnetic responsiveness across all tested loadings (10, 30, and 50 wt%), allowing their straightforward recovery from the aqueous medium, as visually demonstrated in Videos S1–S4. This dual separation capability, combining surface flotation with magnetic attraction and clearly distinguishing the functionalized beads from the unmodified polymer, sets the developed composite apart from conventional suspended photocatalysts, which typically require energy-intensive filtration or centrifugation steps for recovery, and underpins its practical applicability for water/wastewater treatment.

### 3.2. Primary Degradation

**Figure 8:**
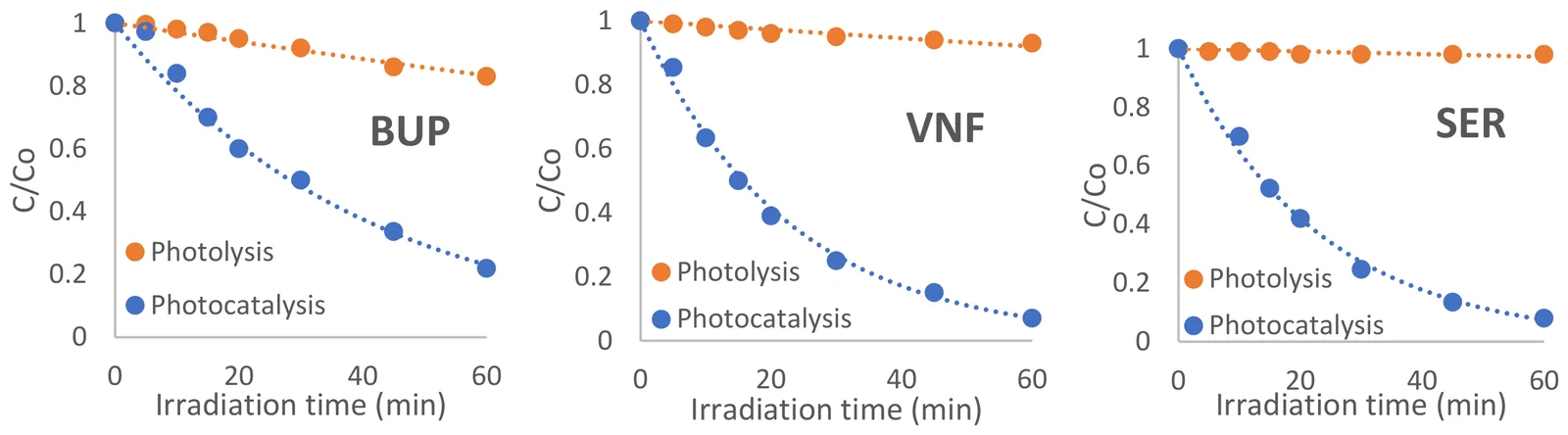
Photolytic and photocatalytic degradation of the selected pharmaceuticals. Catalyst: PET–10% Fe_3_O_4_/TiO_2_, matrix: ultra-pure water, C_0,drug_ = 2 mg/L, C_catalyst_ = 1 g/L.

The photocatalytic activity of the synthesized magnetic beads was initially assessed using the formulation containing the lowest inorganic filler content (10% Fe_3_O_4_/TiO_2_). To establish a baseline for comparison and to evaluate the contribution of irradiation alone, photolytic experiments were conducted in the absence of any catalyst. The results are depicted in Figure 8. As evident from the experimental data, the target pharmaceuticals exhibited limited degradation under simulated solar irradiation. After 60 min of irradiation, degradation percentages of 17%, 7%, and 2% were observed for BUP, VNF, and SER, respectively. These results indicate that photolysis alone was not effective for the removal of the investigated compounds under the applied experimental conditions.

Among the studied pharmaceuticals, BUP showed the highest degradation, while VNF and especially SER remained largely unaffected throughout the irradiation period. Overall, the low degradation efficiencies suggest that the examined compounds are relatively stable under simulated solar irradiation.

Furthermore, any contribution from indirect photolytic processes appears to be limited, as reflected by the low overall removal achieved during the experimental period. Therefore, irradiation alone is insufficient to ensure substantial degradation of these pharmaceuticals [48]. However, in the presence of the catalyst, increased degradation is observed, reaching almost 80% for BUP and more than 90% for VNF and SER within the same time period. This augmentation in their observed degradation can be solely attributed to the catalyst’s efficiency. Finally, blank experiments in the dark showed negligible absorption of the target chemicals onto the synthesized catalyst.

The degradation of the target pharmaceuticals follows the first-order kinetic law. The calculated rate constants obtained from the photolytic and photocatalytic degradation of the drugs are presented in Table 3. Obviously, the reaction rate has a three-fold increase after the application of the catalyst, indicating the efficiency of the synthesized catalyst in degrading the organic molecules.

### 3.3. Effect of Inorganic Content

**Figure 9:**
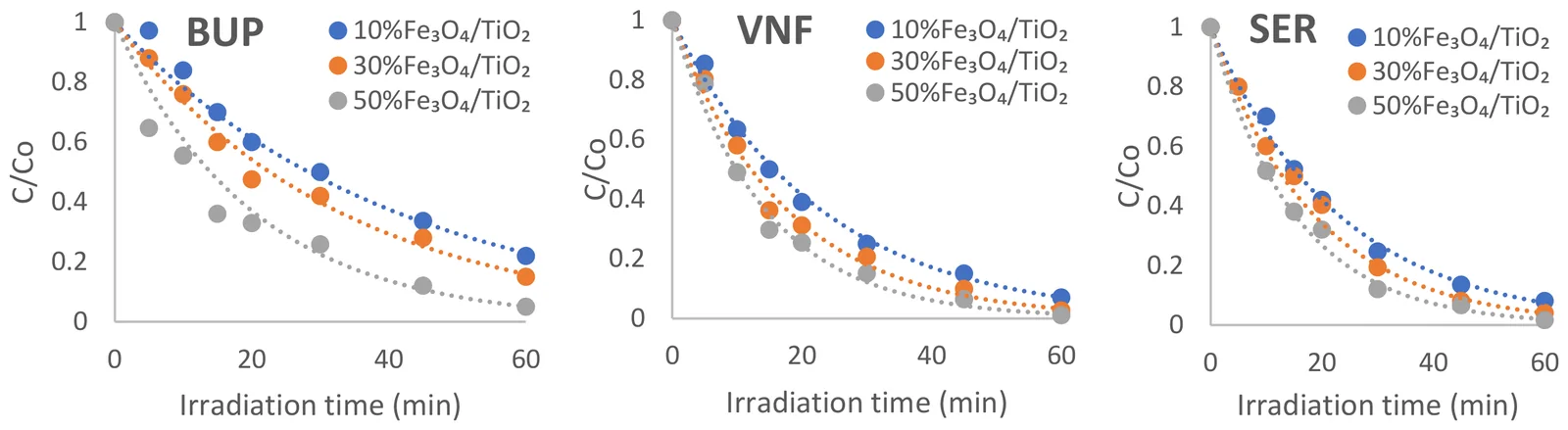
Photocatalytic degradation of the selected pharmaceuticals in the presence of synthesized beads with different inorganic contents. Matrix: ultra-pure water, C_0,drug_ = 2 mg/L, C_catalyst_ = 1 g/L.

To investigate the effect of inorganic content, three different types of synthesized beads were tested for the degradation of the pharmaceutical mixture. The results are presented in Figure 9, while the calculated rate constants are shown in Table 4. As observed, an increase in inorganic content leads to an enhancement in the reaction rate.

For all three target compounds, the catalyst containing 50% Fe_3_O_4_/TiO_2_ exhibited the highest degradation efficiency, achieving up to 95% degradation for BUP and nearly 99% for VNF and SER. Furthermore, the photocatalytic rate constant increased as the inorganic content of the catalyst rose from 10% to 30% and ultimately to 50% for all three antidepressants. This effect was particularly pronounced for BUP, which exhibited a two-fold increase in the presence of the 50% Fe_3_O_4_/TiO_2_ nanocomposite. The improved photocatalytic performance observed at higher Fe_3_O_4_/TiO_2_ contents may be associated with the increased availability of active catalytic sites and the enhanced production of reactive oxygen species during irradiation. In addition, the higher inorganic fraction could facilitate light absorption and promote interfacial photocatalytic reactions, leading to faster degradation kinetics for all three antidepressants [9,31].

### 3.4. Effect of Mass of Catalyst

To investigate the effect of catalyst dosage on the photocatalytic degradation of the three antidepressants, additional experiments were performed using PET/Fe_2_O_3_/TiO_2_ floating beads at concentrations of 500, 1000, and 2000 mg/L. The corresponding degradation rate constants are presented in Table 5. Increasing the catalyst concentration from 500 to 1000 mg/L resulted in an increase in the degradation rates of all three antidepressants, indicating that the higher catalyst loading provided a greater available photocatalytic surface. However, a further increase in catalyst concentration from 1000 to 2000 mg/L resulted in only a marginal improvement in the degradation rates. This behavior may be attributed to the increased amount of floating photocatalytic material in the reaction system, which can partially hinder the penetration and effective distribution of the incident light and increase light scattering. Therefore, a catalyst concentration of 1000 mg/L was selected for the subsequent experiments, as a further increase to 2000 mg/L did not result in a substantial improvement in the degradation kinetics.

### 3.5. Effect of Matrix

**Figure 10:**
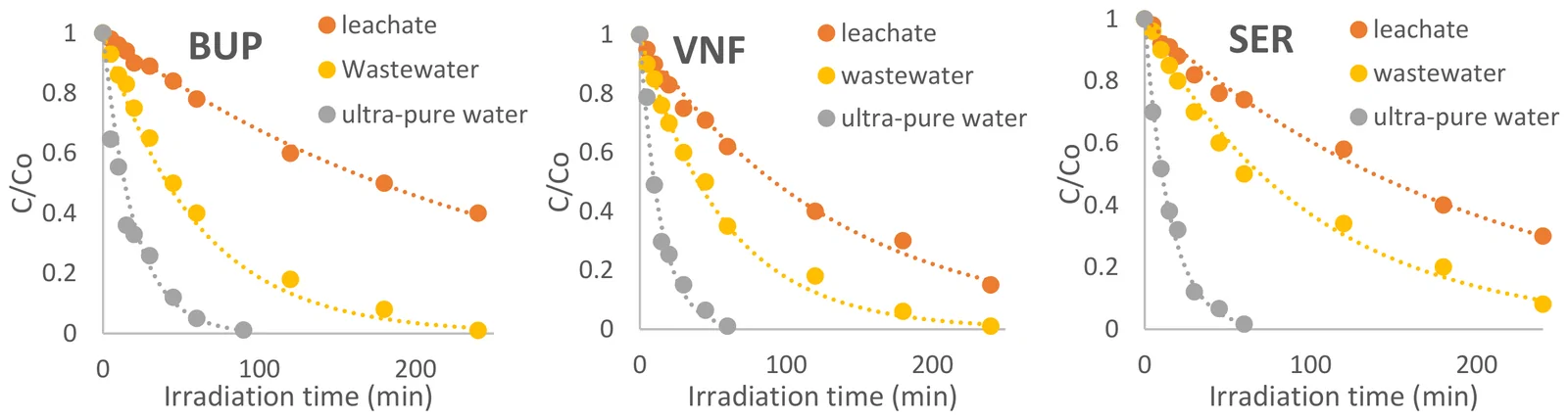
Photocatalytic degradation of the selected pharmaceuticals in different aquatic matrices. Catalyst: PET–50%Fe_3_O_4_/TiO_2_, C_0,drug_ = 2 mg/L, C_catalyst_ = 1 g/L.

Matrix composition was found to have a pronounced effect on the photocatalytic degradation of the investigated compounds (Figure 10 and Table 6). Faster degradation kinetics were observed in ultrapure water, whereas significantly lower removal rates were recorded in wastewater and leachate samples. This difference can be attributed to the distinct physicochemical characteristics of the real water matrices and their higher organic and inorganic load compared with ultrapure water. The effect appears to be particularly pronounced in the leachate, which exhibited a substantially higher overall matrix load than the WWTP effluent. The reduced efficiency in complex matrices is mainly associated with the scavenging of reactive oxygen species by dissolved organic matter, as well as the competitive adsorption of inorganic constituents onto catalyst active sites. Inorganic anions and cations may also interact with the catalyst surface or reactive species, thereby affecting the availability of active sites and reactive species for pollutant degradation [49,50].

In addition to these chemical interactions, physical attenuation of the incident light may also contribute to the lower photocatalytic efficiency. The noticeable coloration of the leachate could reduce light penetration through the solution and consequently limit photon absorption by the photocatalyst [51]. This effect may be particularly relevant for a heterogeneous photocatalytic system, where efficient irradiation of the catalyst surface is required for the generation of electron–hole pairs and subsequent reactive species. The combined effects of radical scavenging, competition for active sites, and light attenuation may therefore explain the substantially slower degradation observed in the real water matrices compared with ultrapure water.

These results highlight the importance of matrix composition in assessing the performance of photocatalytic systems under realistic water-treatment conditions. Although the composite retained photocatalytic activity in the investigated environmental matrices, the lower degradation rates indicate that treatment of complex waters may require longer irradiation times or optimization of the operating conditions to achieve the desired removal [31,52].

### 3.6. Scale-Up Performance

Pilot-scale experiments were conducted to evaluate the photocatalytic performance of magnetic beads in a mixture of three antidepressant compounds. A catalyst dosage of 1000 mg/L was used, corresponding to the optimum concentration determined from the laboratory-scale experiments. The obtained results were found to be generally consistent with those observed at the laboratory scale (Table 7). In addition, the effect of different flow rates was investigated; however, no appreciable differences in photocatalytic performance were observed across the tested conditions. These findings suggest that the system exhibits stable operational behavior under the examined scale-up conditions and variations in hydrodynamic parameters.

### 3.7. Mineralization

**Figure 11:**
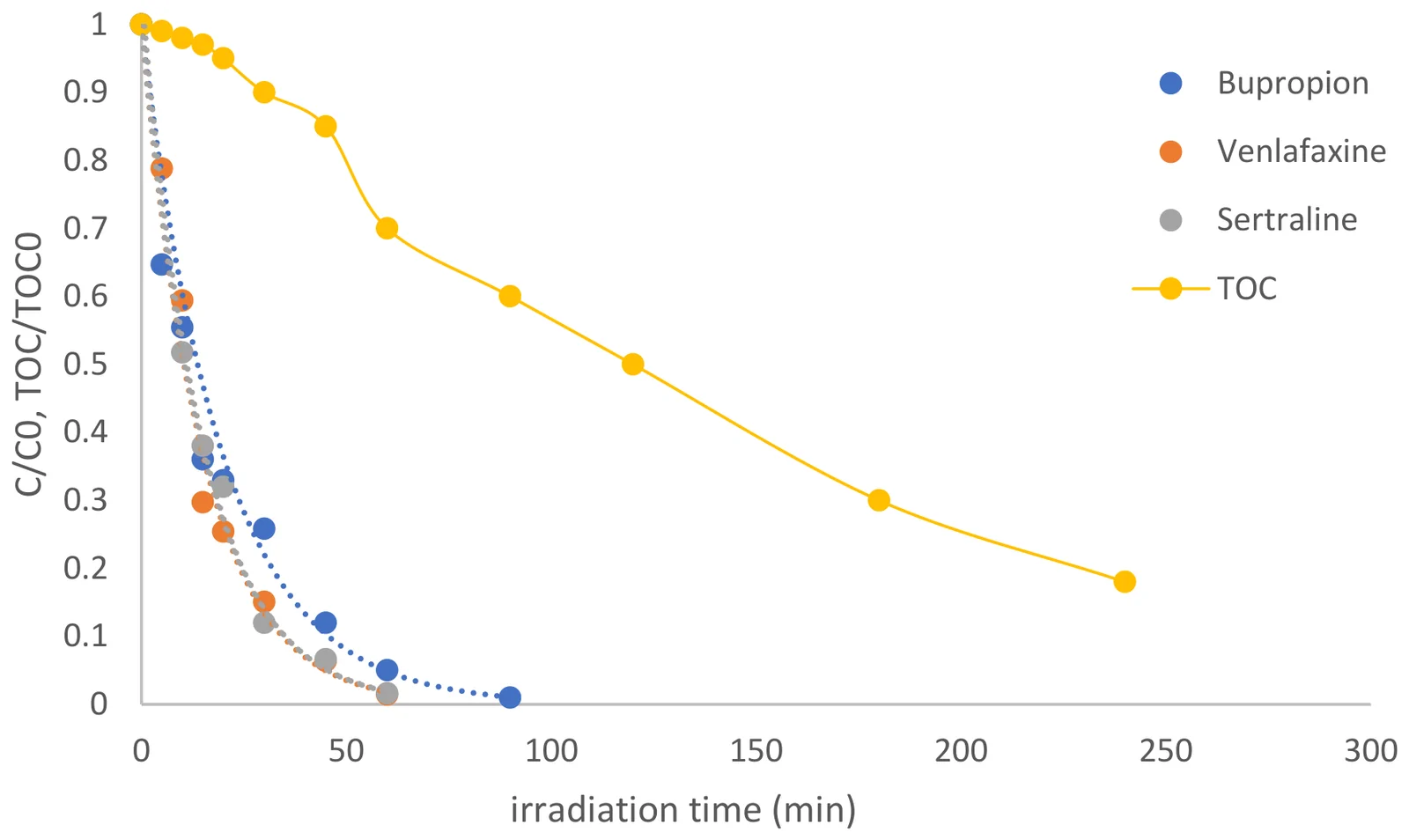
TOC reduction and photocatalytic degradation of the parent compounds during photocatalytic treatment of the mixture. Catalyst: PET–50% Fe_3_O_4_/TiO_2_, C_0,drug_ = 2 mg/L, C_catalyst_ = 1 g/L.

Total organic carbon (TOC) measurements were used to evaluate the extent of mineralization during the photocatalytic treatment (Figure 11). Although rapid degradation of the parent compounds was observed, TOC removal proceeded at a slower rate, indicating that the disappearance of the parent compounds was not accompanied by an equivalent decrease in the total organic carbon content. A gradual decrease in TOC values was recorded throughout the irradiation period, reaching approximately 80% removal after prolonged treatment (4 h). These results suggest that pollutant degradation does not directly correspond to complete mineralization, highlighting the need for extended irradiation times to achieve full conversion of organic carbon into inorganic end products.

### 3.8. Reuse

The potential reusability of the PET–Fe_3_O_4_/TiO_2_ magnetic beads was assessed by conducting two successive photocatalytic cycles. Table 8 presents the calculated rate constants for both cycles in the presence of the three different magnetic beads. The results indicate that the photocatalytic activity of the three catalysts was maintained over the two tested cycles, as the rate constants remained relatively consistent between the first and second cycles. Nevertheless, after the second cycle, deformation of all the composite beads was observed, likely due to the degradation of the polymeric matrix. This phenomenon was more pronounced for the beads with higher inorganic content and is attributed to the reactive oxygen species generated by TiO_2_, which, aside from targeting organic pollutants, may also degrade the surrounding polymeric chains. This observation is consistent with our previous studies, which showed that TiO_2_ starts to wear off after the second cycle, with a significant TiO_2_ mass loss being recorded, particularly for catalysts with higher inorganic content [9,31]. Thus, although the photocatalytic activity was maintained over the two cycles investigated, the observed deformation and TiO_2_ loss indicate that the physical integrity of the composite beads was affected during reuse.

To further assess the effect of reuse on the composite beads, the PET–30% and PET–50% Fe_3_O_4_/TiO_2_ beads were characterized after the second cycle by ATR-FTIR, XRD and SEM–EDS (Figures S4 and S5). The ATR-FTIR spectra of the used beads (Figure S4a) retained all the characteristic PET bands at ~1715 cm^−1^ (C=O stretching), ~1240 and 1100 cm^−1^ (C–O–C stretching of the ester group) and ~725 cm^−1^ (out-of-plane aromatic C–H bending), without noticeable shifts, indicating that the chemical structure of the polymer backbone was largely preserved. However, a broad band appeared at ~3400 cm^−1^, attributed to O–H stretching vibrations, which may be associated with the formation of hydroxyl and carboxyl end groups through partial photo-oxidation of the PET surface by the reactive species generated during treatment, as well as with the higher exposure of the hydroxylated Fe_3_O_4_/TiO_2_ surface. The enhanced absorption in the 800–450 cm^−1^ region, particularly for the PET–50% beads, further suggests that a larger fraction of the inorganic phase was exposed at the bead surface after use. The XRD patterns of the used beads (Figure S4b) retained the characteristic reflections of anatase TiO_2_ and of the magnetite spinel phase, without new reflections attributable to other iron oxide phases. A slight shift in the reflections towards lower 2θ values, observed for the used PET–30% beads, is attributed to sample displacement arising from the irregular surface of the deformed beads rather than to structural changes; moreover, an oxidation of magnetite to maghemite would shift the spinel reflections towards higher angles. Compared with the fresh beads (Figure 3III), the used PET–50% beads exhibited a rougher surface with pronounced wrinkles, folds and loosely attached particles (Figure S5a,b), while EDS mapping showed that Ti and Fe remained homogeneously distributed over the bead surface (Figure S5c–f), indicating that a substantial fraction of the Fe_3_O_4_/TiO_2_ particles was retained. Overall, these results indicate that the deformation observed after the second cycle originates mainly from the surface oxidation and partial erosion of the PET matrix rather than from structural changes in the embedded photocatalyst, in agreement with the retained photocatalytic activity (Table 8). At the same time, the progressive erosion of the polymer surface can account for the gradual detachment of inorganic particles and the release of Fe species into the solution (Section 3.9), which represents the main limitation for the long-term reuse of the beads.

### 3.9. Mechanistic Considerations

To experimentally investigate the contribution of the main reactive species involved in the photocatalytic degradation process, additional radical scavenging experiments were performed. Isopropanol, potassium iodide (KI), and benzoquinone were used as scavengers of hydroxyl radicals (^•^OH), photogenerated holes (h^+^), and superoxide radicals (O_2_^•−^), respectively. The addition of the scavengers resulted in reductions in the degradation rate of approximately 78%, 87%, and 64%, respectively. The strongest inhibition was observed in the presence of KI, followed by isopropanol and benzoquinone, indicating that photogenerated holes and ^•^OH played important roles in the degradation process, while O_2_^•−^ also contributed to the overall oxidation of the antidepressant compounds. These results support the involvement of the investigated reactive species in the photocatalytic degradation process.

The photocatalytic performance of a semiconductor like TiO_2_ relies on absorbing light energy that exceeds its band gap energy. As a result, electrons (e^−^) in the valence band become excited and transition to the conduction band, leaving behind holes (h^+^) in the valence band. The generated electron–hole pairs may either recombine within a few nanoseconds, returning to their original state, or remain active and engage in redox reactions. During this process, they can generate reactive species such as hydroxyl radicals (OH^•^), superoxide anion radicals (O_2_^•−^), hydroperoxyl radicals (HO_2_^•^), and hydrogen peroxide (H_2_O_2_). These reactive oxygen species, along with the holes, play a crucial role in oxidative pathways that contribute to the degradation of co-existing pollutants (Figure S6) [53,54].

TiO_2_ + hv → h^+^_vb_ + e^−^_cb_

O_2_ + e^−^ → O_2_^●−^

H_2_O + h^+^ → ^●^OH + H^+^

Fe_3_O_4_ may also contribute to the photocatalytic degradation process through its Fe^2+^/Fe^3+^ redox chemistry. In particular, the Fe^2+^/Fe^3+^ redox pair associated with Fe_3_O_4_ can participate in Fenton and Fenton-like reactions, contributing to the formation of hydroxyl radicals and potentially promoting the degradation of organic contaminants. In addition, Fe_3_O_4_ has been reported to exhibit light absorption at higher wavelengths, as also observed in the DRS spectrum of the hybrid particles (Figure S2b), which may further contribute to the photocatalytic behavior of the composite (Figure S6) [55].

2O_2_^●−^ + 2H^+^ → H_2_O_2_ + O_2_

Fe^2+^ + H_2_O_2_ → Fe^3+^+ ^●^OH + OH^−^

Iron release measurements in the treated solutions further support the possible contribution of Fenton-related pathways. The detected iron concentrations increased with increasing inorganic content of the nanocomposite, reaching 0.125 mg/L, 0.301 mg/L, and 0.522 mg/L for PET–10% Fe_3_O_4_/TiO_2_, PET–30% Fe_3_O_4_/TiO_2_, and PET–50% Fe_3_O_4_/TiO_2_, respectively. This trend suggests that higher Fe-containing catalyst loading may increase the availability of Fe species capable of participating in Fenton-related reactions, thereby potentially contributing to the enhanced degradation efficiency observed. However, the present experimental design does not allow the relative contribution of dissolved Fe-mediated reactions to be distinguished from that of the heterogeneous photocatalytic processes.

## 4. Conclusions

In this study, a novel floating magnetic PET–Fe_3_O_4_/TiO_2_ composite photocatalyst was successfully developed for the efficient removal of antidepressant contaminants from aqueous matrices under simulated solar irradiation. The integration of magnetic Fe_3_O_4_/TiO_2_ nanoparticles onto recycled PET beads provided a multifunctional material combining high photocatalytic activity with facile separation, buoyancy, and reusability. The optimized composite containing 50 wt% Fe_3_O_4_/TiO_2_ exhibited the highest degradation performance, achieving rapid removal of venlafaxine, bupropion, and sertraline within 60–90 min.

The enhanced photocatalytic activity of the composite can be attributed to the synergistic contribution of TiO_2_ photocatalysis and iron oxide-mediated redox processes. The incorporation of Fe_3_O_4_ extends the light absorption of the material into the visible region and may provide additional pathways for reactive oxygen species generation through Fe^2+^/Fe^3+^ redox cycling and Fenton-like reactions. These processes contribute to the formation of highly reactive species, such as hydroxyl radicals (^•^OH), which play a key role in the degradation of pharmaceutical compounds. Furthermore, immobilization onto the PET matrix provided a floating and magnetically recoverable composite configuration, facilitating catalyst separation and reuse.

The degradation efficiency was affected by the composition of the aqueous matrix, with reduced performance in complex environmental samples due to radical scavenging and competitive interactions. Although complete elimination of the parent pharmaceuticals was achieved, prolonged irradiation was required for extensive mineralization, indicating that degradation of the parent compounds was not directly accompanied by complete mineralization. The successful operation of the composite in a continuous-flow pilot reactor, together with the retention of photocatalytic activity over the two tested reuse cycles, demonstrates the applicability of the material under the investigated conditions. However, deformation of the composite beads and Fe leaching were observed during reuse, particularly at higher Fe_3_O_4_ contents. The detected Fe concentrations reached 0.522 mg/L, indicating that Fe release should be considered when assessing the environmental applicability of the composite. Although the leached Fe may contribute to Fenton-related reactions, the relative contribution of dissolved iron-mediated and heterogeneous photocatalytic processes could not be quantitatively distinguished in the present study.

Overall, the developed floating magnetic PET–Fe_3_O_4_/TiO_2_ composite represents a sustainable strategy for pharmaceutical removal, combining advanced photocatalytic oxidation, magnetic recoverability, catalyst stability, and the valorization of recycled PET waste. These characteristics demonstrate its potential as an environmentally friendly and scalable material for the remediation of emerging contaminants in water systems.

## Figures and Tables

**Table 1:** Thermal properties of neat PET and PET–Fe_3_O_4_/TiO_2_ composite beads determined by DSC and TGA, including melting temperature (T_m_), crystalline fraction during melting (CF_m_), maximum degradation temperature (T_DTG,max_), and residual mass at 600 °C. The enthalpy values are expressed as relative crystalline fractions.

Sample	T_m_ (°C)	CF_m_ (%)	T_DTG,max_ (°C)	Residue at 600 °C (%)
PET neat	247.9	32.5	448	12.6
PET-Fe_3_O_4_/TiO_2_ 10 wt%	247.8	29.2	448	28.1
PET-Fe_3_O_4_/TiO_2_ 30 wt%	245.9	35.1	447	41.3
PET-Fe_3_O_4_/TiO_2_ 50 wt%	246.6	45.4	448	51.2

**Table 2:** Thermal transitions of the PET composites during the second heating scan (quenched samples) and cooling.

Sample Name	Second Heating					Cooling	
	T_g_ (°C)	T_cc_ (°C)	CF_cc_ (%)	T_m_ (°C)	CF_m_ (%)	T_c_ (°C)	CF_c_ (%)
PET neat	80.6	147.9	28.9	253.7	31.9	188.1	29.1
PET-Fe_3_O_4_/TiO_2_ 10 wt%	78.4	132.9	28.9	247.1	30.1	177.4	26.8
PET-Fe_3_O_4_/TiO_2_ 30 wt%	79.7	133.5	32.5	247.9	34.2	185.4	33.6
PET-Fe_3_O_4_/TiO_2_ 50 wt%	80.2	134.9	36.3	248.8	39.2	190.1	40.5

The enthalpy values are expressed as relative crystalline fractions. CF_cc_ stands for the crystalline fraction during cold crystallization, CF_m_ stands for the crystalline fraction during melting and CF_c_ stands for the crystalline fraction during melt crystallization.

**Table 3:** Photolytic and photocatalytic rate constants of the selected drugs. Catalyst: PET-10%Fe_3_O_4_/TiO_2_, matrix: ultra-pure water, C_0,drug_ = 2 mg/L, C_catalyst_ = 1 g/L.

	BUP		VNF		SRL	
	k (min^−1^)	R^2^	k (min^−1^)	R^2^	k (min^−1^)	R^2^
Photolysis	0.003	0.98	0.0014	0.98	0.0005	0.97
Photocatalysis	0.025	0.99	0.044	0.99	0.043	0.99

**Table 4:** Photocatalytic rate constants of the selected drugs in the presence of the three catalysts. Catalyst: PET–Fe_3_O_4_/TiO_2_, matrix: ultra-pure water, C_0,drug_ = 2 mg/L, C_catalyst_ = 1 g/L.

Catalyst	BUP k (min^−1^)	VNF k (min^−1^)	SRL k (min^−1^)
10% Fe_3_O_4_/TiO_2_	0.025	0.044	0.043
30% Fe_3_O_4_/TiO_2_	0.031	0.057	0.054
50% Fe_3_O_4_/TiO_2_	0.05	0.07	0.066

**Table 5:** Photocatalytic rate constants of the selected drugs under different concentrations of the catalyst. Catalyst: PET–50%Fe_3_O_4_/TiO_2_, matrix: ultra-pure water, C_0,drug_ = 2 mg/L.

Concentration of Catalyst	BUP k (min^−1^)	VNF k (min^−1^)	SRL k (min^−1^)
500 mg/L	0.032	0.051	0.05
1000 mg/L	0.05	0.07	0.066
2000 mg/L	0.055	0.071	0.068

**Table 6:** Photocatalytic rate constants of the selected drugs in different aquatic matrices. Catalyst: PET–50% Fe_3_O_4_/TiO_2_, C_0,drug_ = 2 mg/L, C_catalyst_ = 1 g/L.

Catalyst	BUP k (min^−1^)	VNF k (min^−1^)	SRL k (min^−1^)
Ultrapure Water	0.05	0.07	0.066
Wastewater	0.017	0.018	0.01
Leachate	0.004	0.008	0.005

**Table 7:** Photocatalytic rate constants (k, min^−1^) of the selected antidepressants in the pilot-scale photoreactor at different flow rates.

	BUP k (min^−1^)	VNF k (min^−1^)	SRL k (min^−1^)
1 L/min	0.049	0.068	0.060
5 L/min	0.048	0.071	0.062
10 L/min	0.051	0.070	0.062

**Table 8:** Photocatalytic rate constants (k, min^−1^) of the selected antidepressants during the first and second photocatalytic cycles for all catalysts.

Catalyst	BUP k (min^−1^)		VNF k (min^−1^)		SRL k (min^−1^)	
	1^st^ Cycle	2^nd^ Cycle	1^st^ Cycle	2^nd^ Cycle	1^st^ Cycle	2^nd^ Cycle
10% Fe_3_O_4_/TiO_2_	0.025	0.025	0.044	0.044	0.043	0.042
30% Fe_3_O_4_/TiO_2_	0.031	0.03	0.057	0.056	0.054	0.052
50% Fe_3_O_4_/TiO_2_	0.05	0.048	0.07	0.069	0.066	0.065

## Data Availability

Data are contained within the article or Supplementary Material.

## Supplementary Materials

The following supporting information can be downloaded at https://www.mdpi.com/article/10.3390/polym18202555/s1, Figure S1: Schematic diagram of the pilot-scale UV photocatalytic unit; Figure S2: N_2_ adsorption–desorption isotherm, UV–Vis DRS spectrum and Tauc plot of the Fe_3_O_4_/TiO_2_ particles; Figure S3: SEM–EDS characterization of the hybrid Fe_3_O_4_/TiO_2_ particles; Figure S4: ATR-FTIR spectra and XRD patterns of the composite beads before and after reuse; Figure S5: SEM–EDS analysis of the PET–50 wt% Fe_3_O_4_/TiO_2_ beads after reuse; Figure S6: Reactions taking part in the photocatalytic process; Video S1: Magnetic response test of neat PET beads (negative control); Video S2: Magnetic response test of PET–10 wt% Fe_3_O_4_/TiO_2_ beads; Video S3: Magnetic response test of PET—30 wt% Fe_3_O_4_/TiO_2_ beads; Video S4: Magnetic response test of PET—50 wt% Fe_3_O_4_/TiO_2_ beads.
