# Supplementary material for: From Plastic Waste to Water Treatment: Floating Magnetic Poly(ethylene Terephthalate)–Fe3O4/TiO2 Photocatalysts for Antidepressant Removal
Source: Polymers (Basel). 2026 Oct 17;18(20):2555. doi: 10.3390/polym18202555 (PMC13709069; doi:10.3390/polym18202555)
Supplement: Supplementary file 1 [file polymers-18-02555-s001.zip › Supplementary material.pdf]

polymers

MDPI

Supplementary material for

From Plastic Waste to Water Treatment: Floating Magnetic Poly(ethylene terephthalate)–Fe_3O_4/TiO_2 Photocatalysts for Antidepressants Removal

Eleni Evgenidou [1,2], Nina Maria Ainali [1,3], Androniki Rapti [3], Dimitrios N. Bikiaris [3*], Dimitra A. Lambropoulou [1,2*]

[1] Laboratory of Environmental Pollution Control, Department of Chemistry, Aristotle University of Thessaloniki, GR–541 24 Thessaloniki, Greece,
[2] Centre for Interdisciplinary Research and Innovation (CIRI-AUTH), Balkan Centre, GR-570 01 Thessaloniki, Greece
[3] Laboratory of Polymer & Color Chemistry and Technology, Department of Chemistry, Aristotle University of Thessaloniki, GR-541 24 Thessaloniki, Greece
* Correspondence: dbic@chem.auth.gr (D.N.B); dlambro@chem.auth.gr (D.A.L)

Section S1. Pilot-scale photocatalytic unit

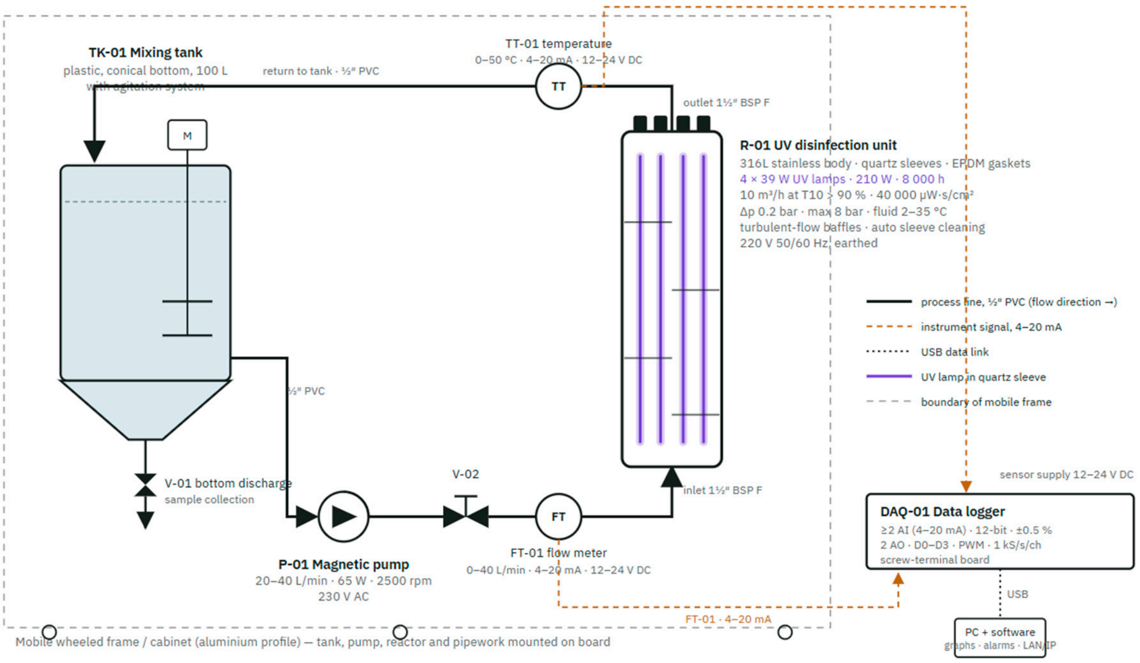

Flow path: TK-01 → P-01 → V-02 → FT-01 → R-01 (bottom inlet, top outlet) → TT-01 → back to TK-01. The loop is closed, so the batch in the tank is recirculated through the UV unit for the required number of passes; V-01 drains the tank for sampling. FT-01 and TT-01 feed the two 4–20 mA inputs of DAQ-01. Tag numbers are assigned here for reference and do not appear in the specification.

Figure S1. Schematic diagram of the laboratory pilot Unit- UV photocatalytic reactor.

Section S2. Characterization of the Fe_3O_4/TiO_2 particles

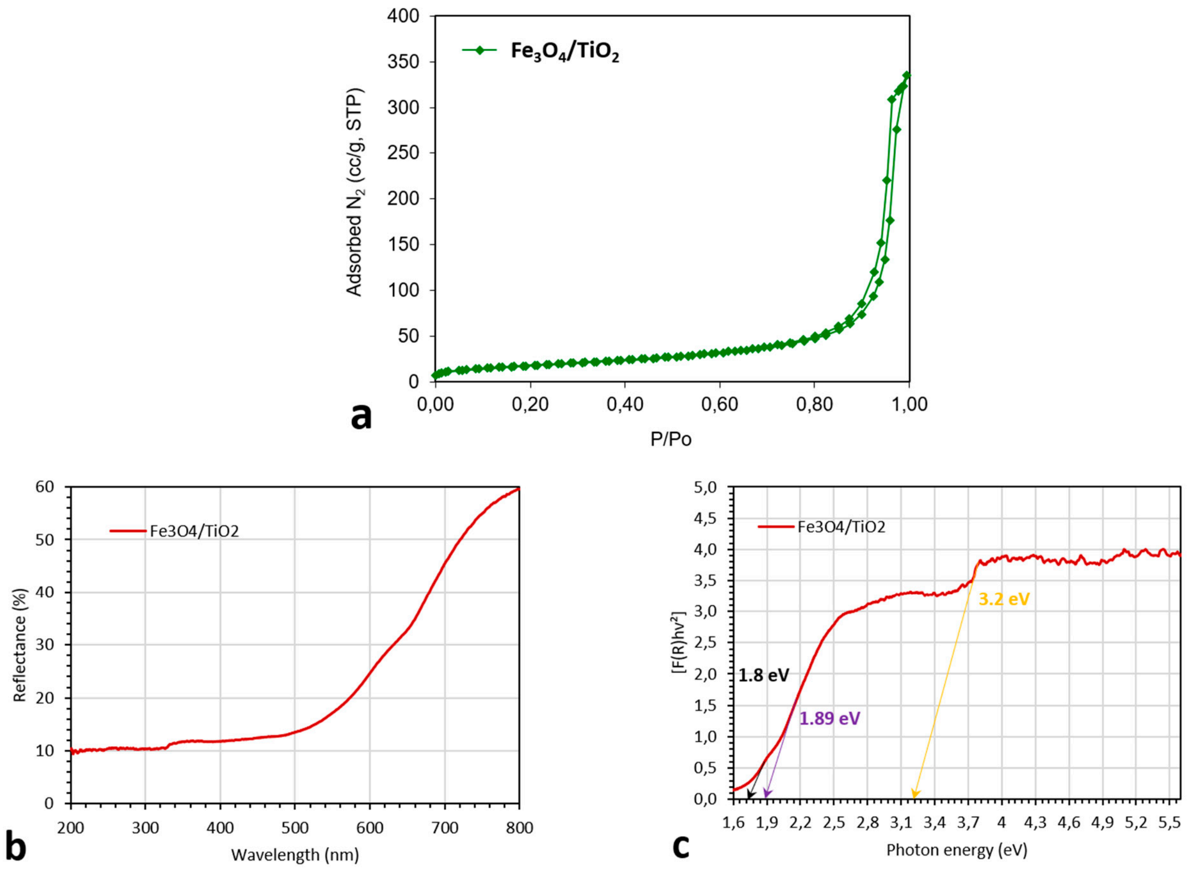

Figure S2. (a) N_2 adsorption–desorption isotherm of the Fe_3O_4/TiO_2 nanoparticles (S_BET = 65 m^2 g^{-1}, total pore volume = 0.519 cm^3 g^{-1} at P/P_0 = 0.99); (b) UV–Vis diffuse reflectance spectrum and (c) corresponding Tauc plot, $[F(R)h\nu]^2$ versus photon energy, of the Fe_3O_4/TiO_2 nanoparticles.

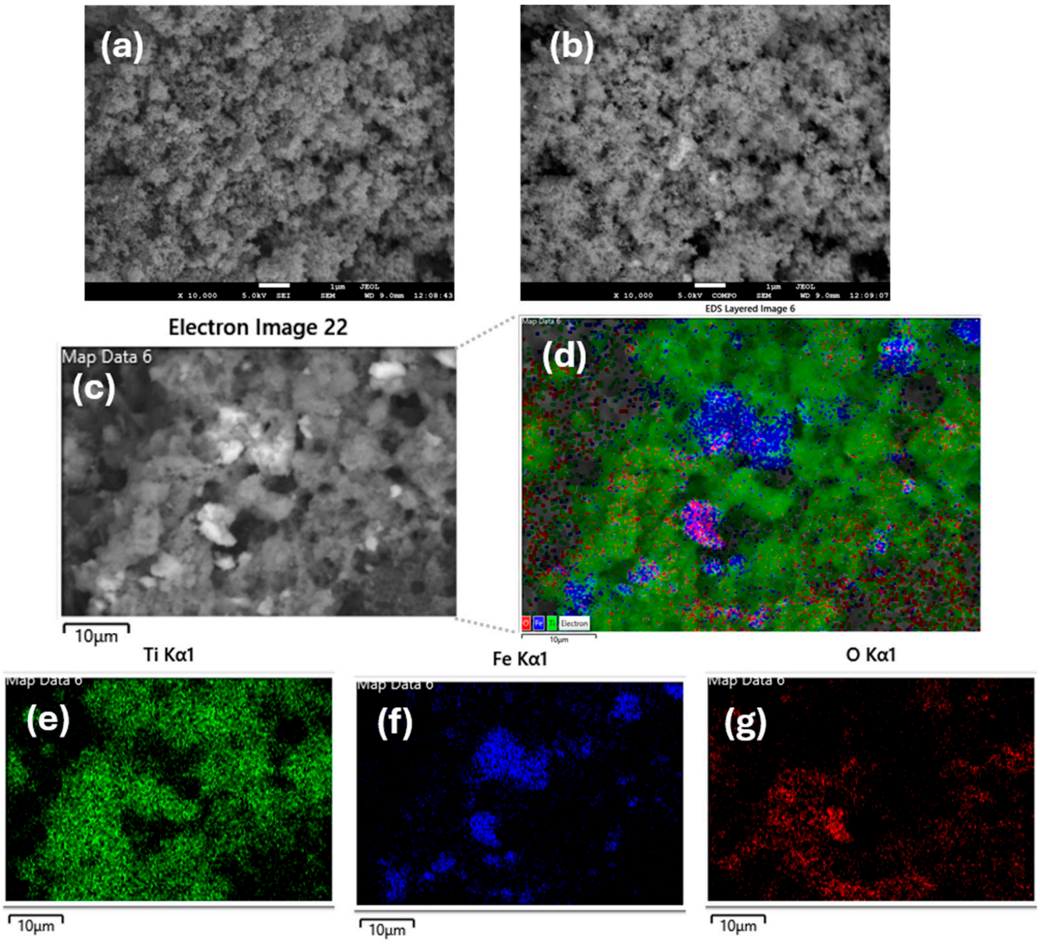

Figure S3. SEM-EDS characterization of the hybrid Fe_3O_4/TiO_2 particles: (a) secondary electron and (b) back-scattered electron images (×10,000, 5 kV); (c) electron image of a different area selected for elemental mapping and (d) the corresponding overlay EDS map of O (red), Fe (blue) and Ti (green); individual elemental maps of (e) Ti, (f) Fe and (g) O. The elemental composition determined from the map sum spectrum was O 36.1 wt%, Ti 46.6 wt% and Fe 17.4 wt%.

Section S3. Characterization of the composite beads after reuse

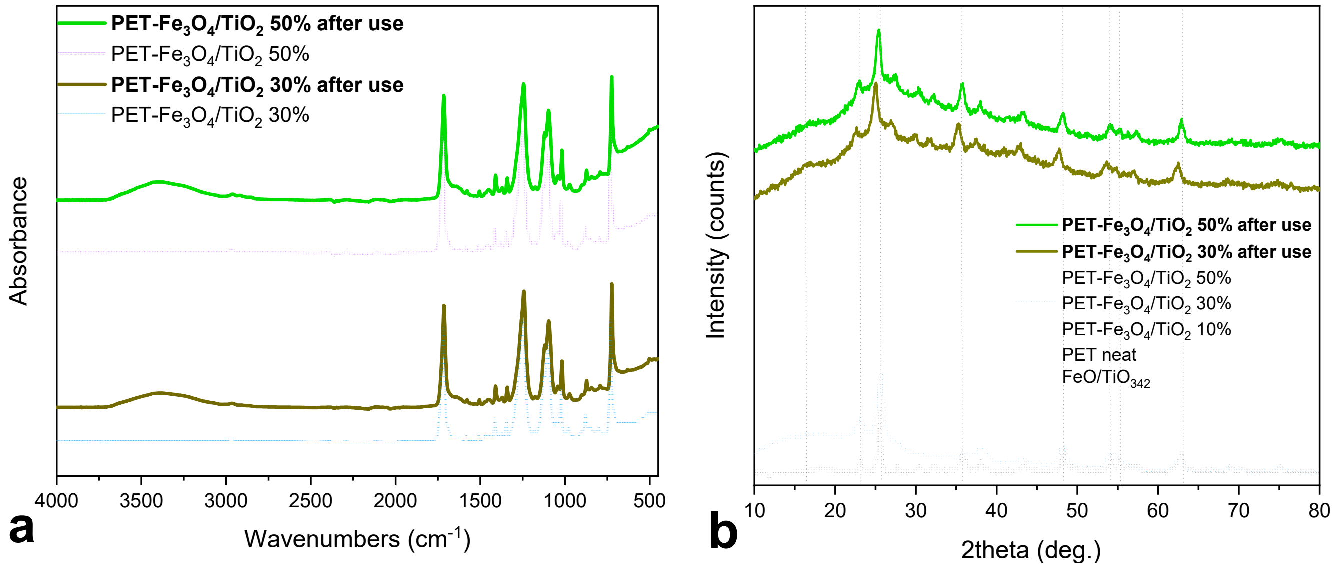

Figure S4. (a) ATR-FTIR spectra and (b) XRD patterns of the PET–Fe_3O_4/TiO_2 30 wt% and 50 wt% composite beads before and after the second photocatalytic cycle. The XRD patterns of neat PET, the PET–Fe_3O_4/TiO_2 10 wt% beads and the Fe_3O_4/TiO_2 nanoparticles are included for comparison.

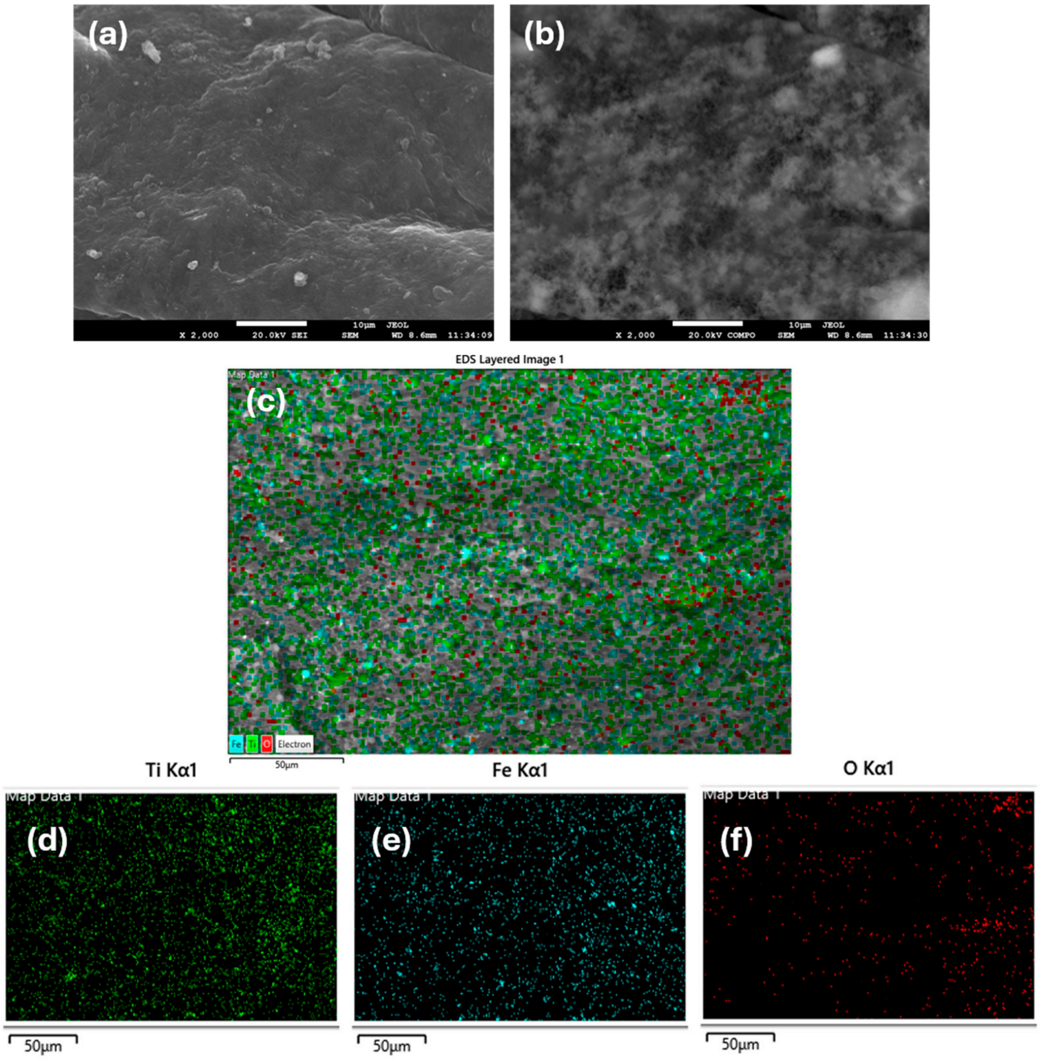

Figure S5. SEM–EDS analysis of the PET–Fe_3O_4/TiO_2 50 wt% composite beads after the second photocatalytic cycle: (a) secondary electron (SE) image showing the surface morphology; (b) back-scattered electron (BSE) image showing the distribution of the Fe_3O_4/TiO_2 particles (bright regions); (c) overlay EDS elemental map of Fe (cyan), Ti (green) and O (red) on the corresponding electron image; and individual elemental maps of (d) Ti, (e) Fe and (f) O.

Section S4. Proposed photocatalytic mechanism

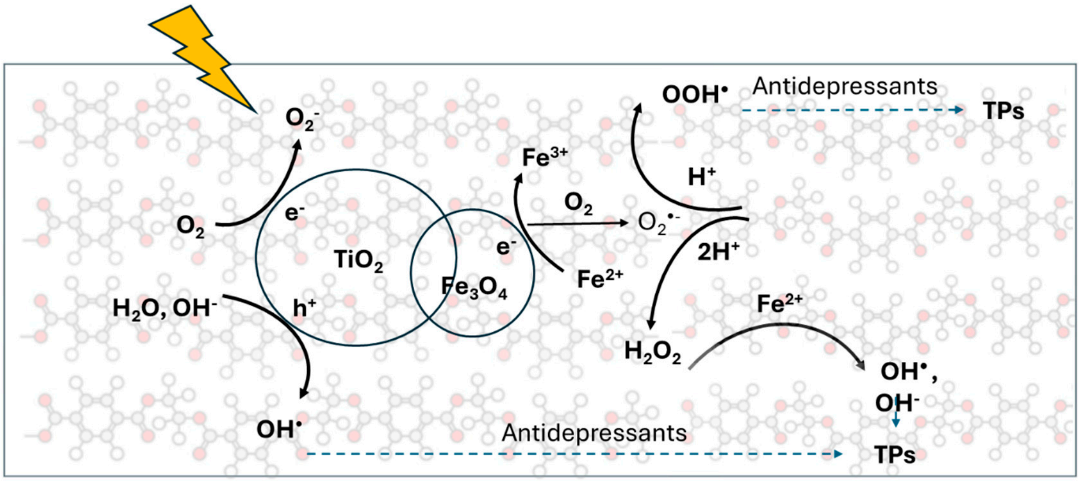

Figure S6. Reactions taking part in the photocatalytic process with the magnetic PET-Fe_3O_4/TiO_2 beads.
